# Supplementary material for: Multiplexed functional metagenomic analysis of the infant microbiome identifies effectors of NF-κB, autophagy, and cellular redox state
Source: Cell Rep. 2021 Sep 21;36(12):109746. doi: 10.1016/j.celrep.2021.109746 (PMC8480279; doi:10.1016/j.celrep.2021.109746)
Supplement: Document S1. Figures S1–S4 [file mmc1.pdf]

Cell Reports, Volume *36*

Supplemental information

Multiplexed functional metagenomic analysis of the infant microbiome identifies effectors of NF-κB, autophagy, and cellular redox state

Frank J. Piscotta, Shawn T. Whitfield, Toshiki G. Nakashige, Andreia B. Estrela, Thahmina Ali, and Sean F. Brady

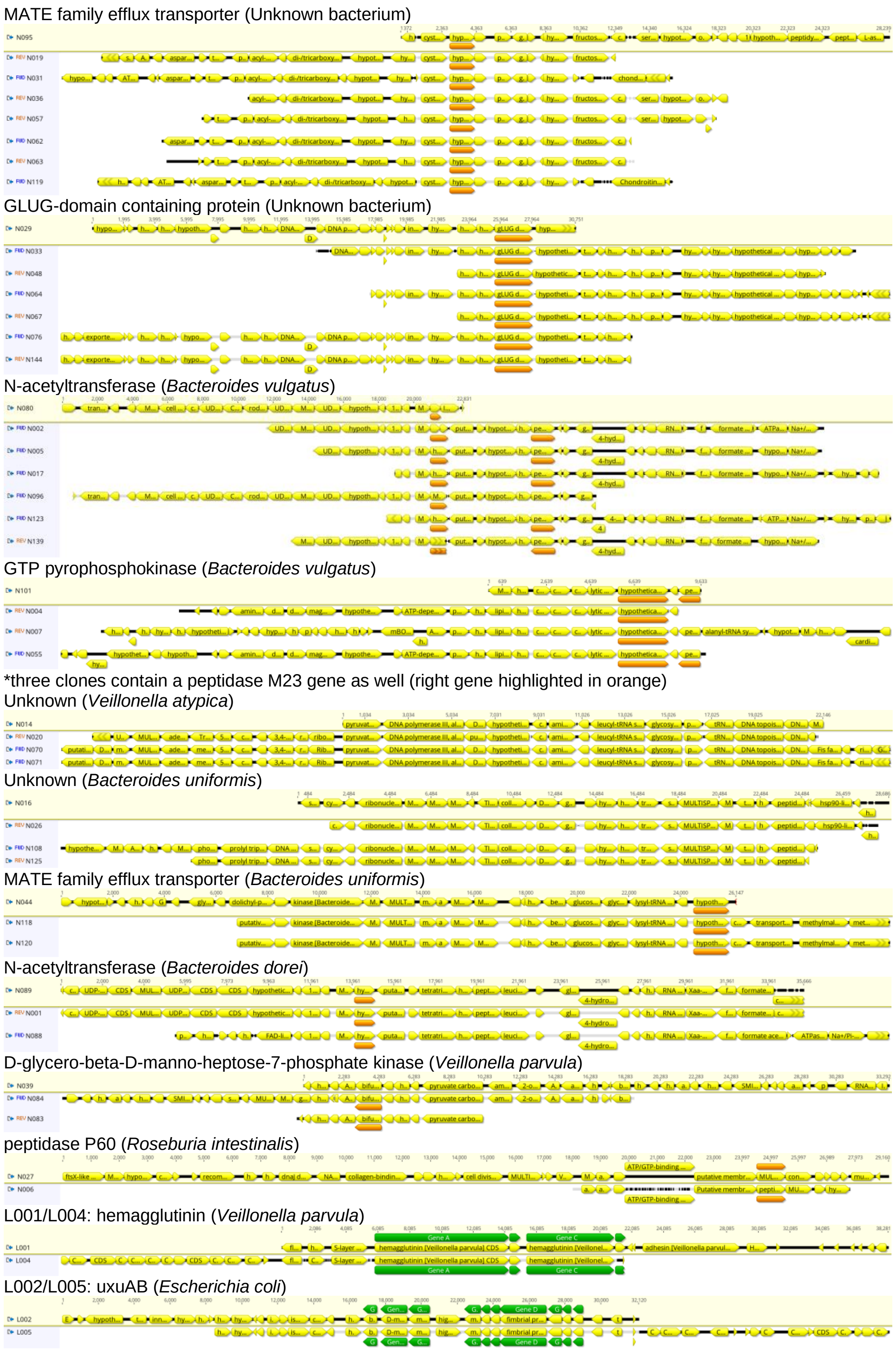

Figure S1 Overlapping genomic regions obtained by alignment of sequenced bioactive cosmids. Orange highlighting indicates genes with known NF-κB-inducing activity or genes suggested to be bioactive by transposon mutagenesis. Green highlighting indicates prospective autophagy-inducing genes selected for subcloning in Figure 4. Related to Figure 3C-D, Figure 4A.

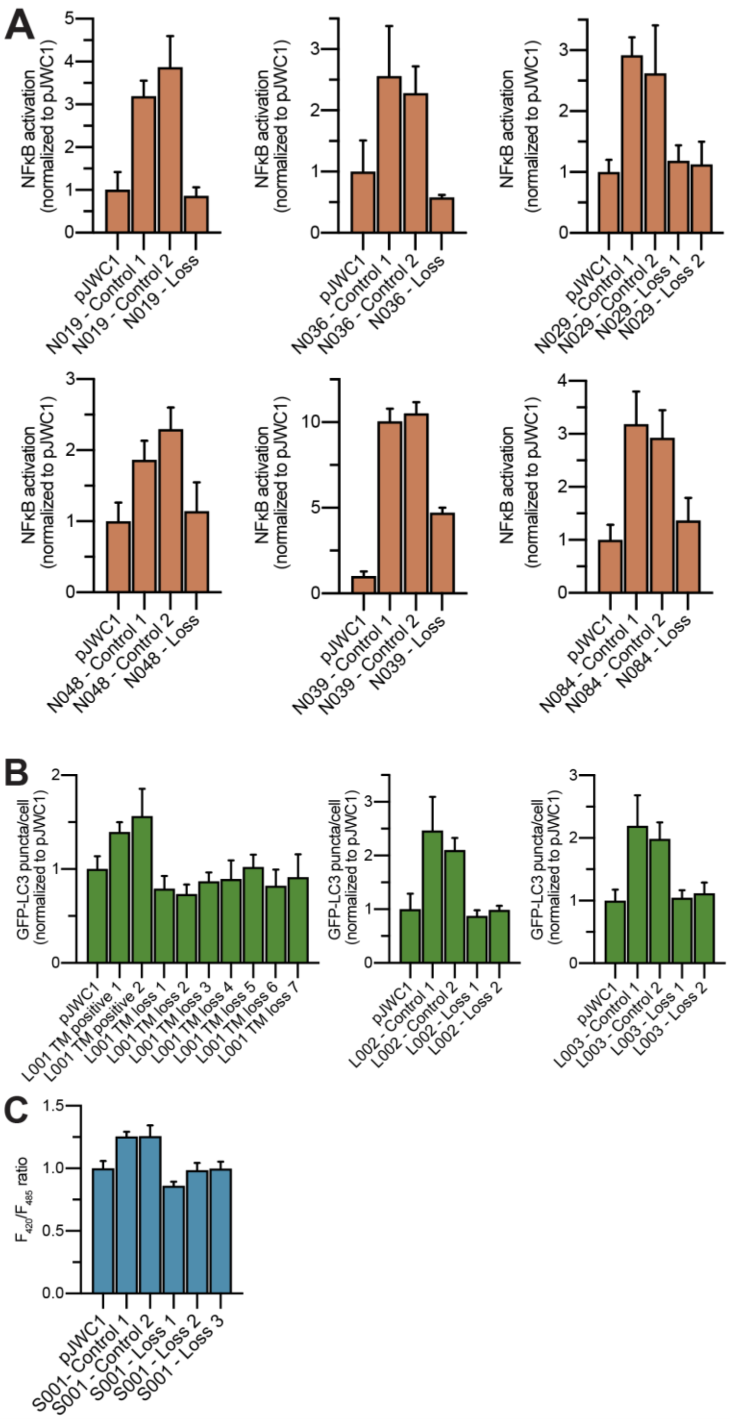

Figure S2 Transposon mutagenesis of selected cosmid clones for identification of genes causing activation of NF-κB, LC3 or SoNar reporter lines. All data are represented as mean ± SD, n=4. (A) Transposon mutagenesis of NF-κB active clones. “Loss” samples indicate where transposon insertions resulted in loss of activity, as confirmed by Sanger sequencing. Related to Figure 3D. (B) Transposon mutagenesis of autophagy active clones. “Loss” samples indicate where transposon insertions resulted in loss of activity, as confirmed by Sanger sequencing. Related to Figure 4A. (C) Transposon mutagenesis of SoNar active clones. “Loss” samples indicate where transposon insertions resulted in loss of activity, as confirmed by Sanger sequencing. Related to Figure 5A.

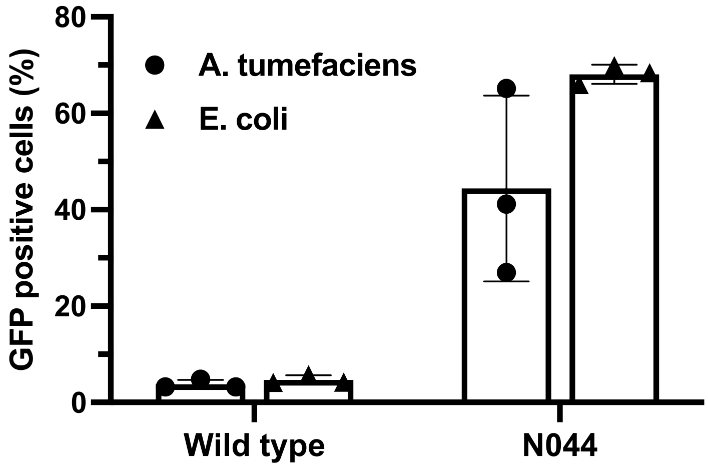

Figure S3. *A. tumefaciens* (circle) and *E. coli* (triangle) transformed with the MATE family exporter-carrying cosmid N044 activate NF-kB reporter cells. Both wild-type strains and strains transformed with N044 were grown at 30 °C for 2 d and supernatants collected by centrifugation were applied to NF-kB reporter cells at a 1:5 dilution. All data are represented as mean ± SD, n=3. Related to Figure 3.

N-acetyltransferase (*Bacteroides vulgatus*)

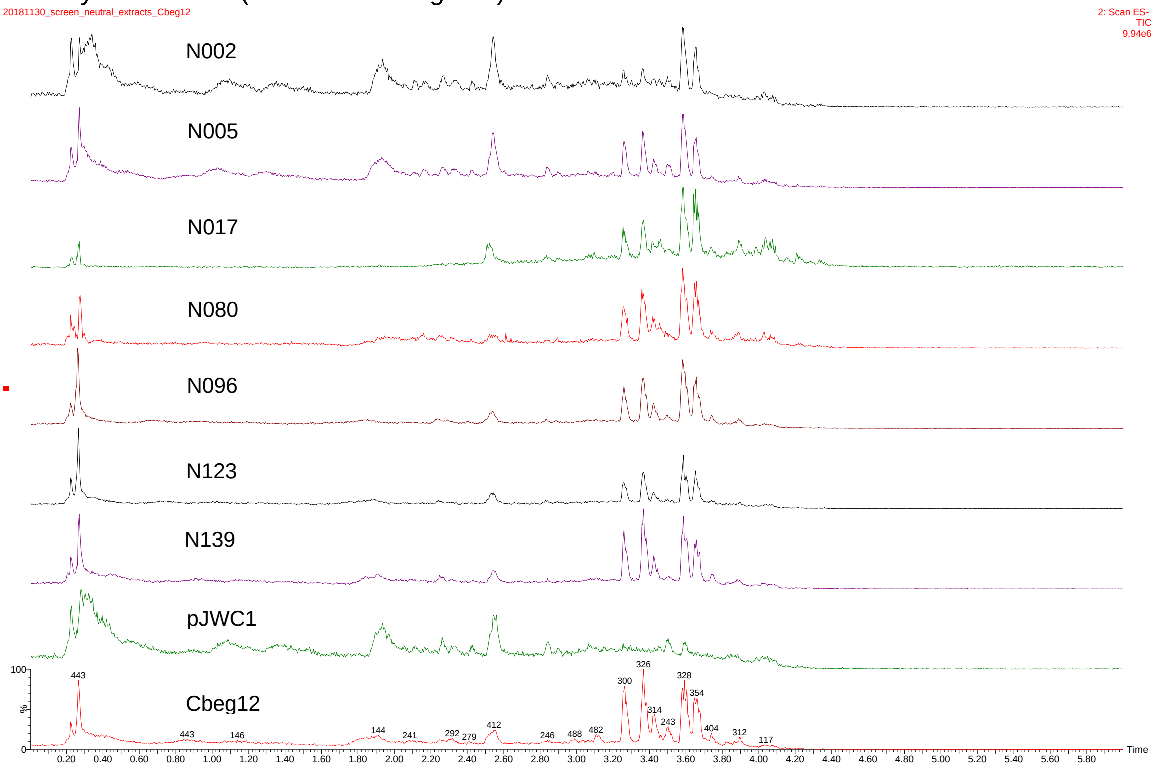

N-acetyltransferase (*Bacteroides dorei*)

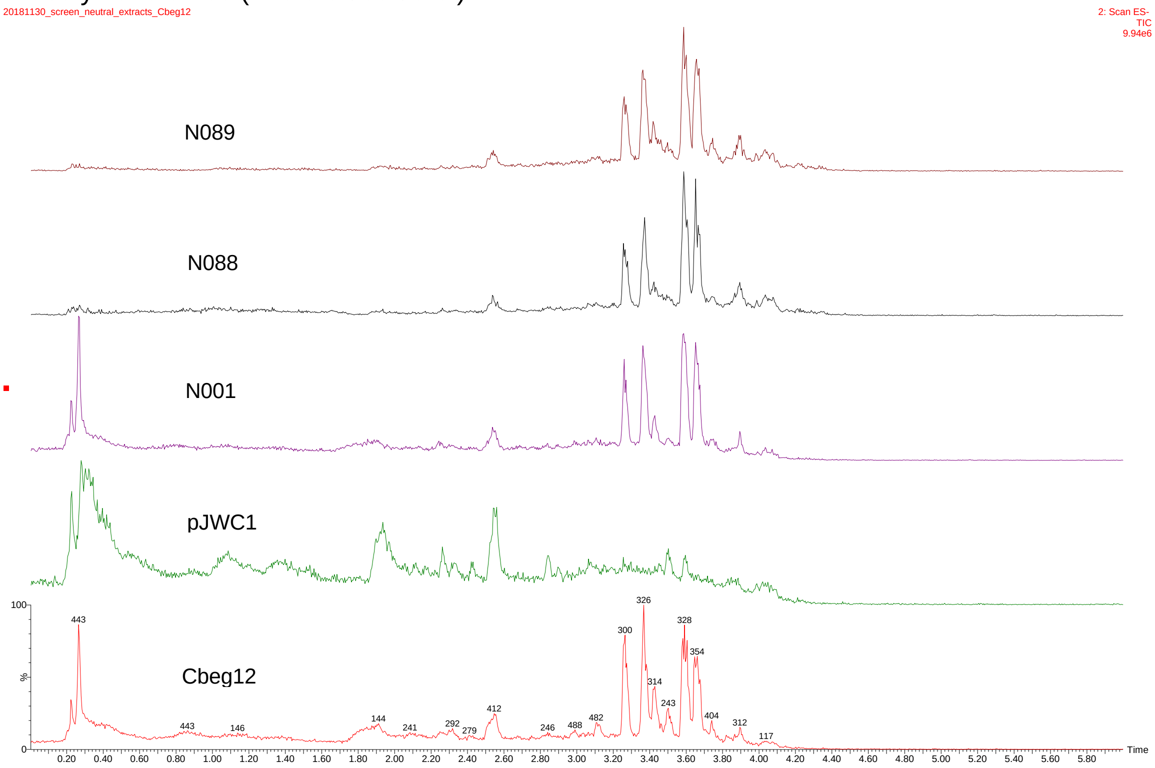

Figure S4 LCMS traces of extracts from commendamide-producing N-acetyltransferase clones compared to pJWC1 empty control and Cbeg12 positive (commendamide-producing) control. Commendamide-related products of masses of 300, 325, 328 and 354 are found in all positive samples and Cbeg12 but not pJWC1. Related to Figure 3E.
