# Supplementary material for: Multiplexed functional metagenomic analysis of the infant microbiome identifies effectors of NF-κB, autophagy, and cellular redox state
Source: Cell Rep. 2021 Sep 21;36(12):109746. doi: 10.1016/j.celrep.2021.109746 (PMC8480279; doi:10.1016/j.celrep.2021.109746)
Supplement: Document S2. Article plus supplemental information [file mmc7.pdf]

Cell Reports

Article

Multiplexed functional metagenomic analysis of the infant microbiome identifies effectors of NF-κB, autophagy, and cellular redox state

Graphical abstract

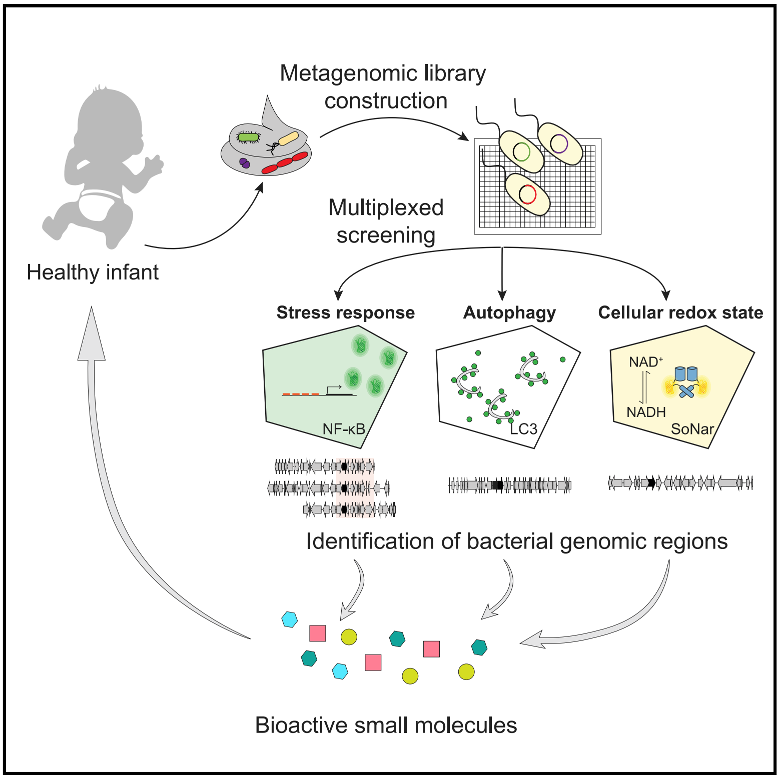

Authors

Frank J. Piscotta, Shawn T. Whitfield, Toshiki G. Nakashige, Andreia B. Estrela, Thahmina Ali, Sean F. Brady

Correspondence

sbrady@rockefeller.edu

In brief

Piscotta et al. screen a metagenomic library constructed from 14 infant stool microbiomes for effectors of host cell immunity, autophagy, and redox potential. The bioactivities they identify from the infant microbiome expand our understanding of microbial effectors that are known to modulate host pathways.

Highlights

- Construction of a metagenomic library from stool of infants
- A multiplexed screen for bacterial effectors of host cellular processes
- Identification of microbiome-encoded effectors hydrogen sulfide and commendamide
- The products of a *Klebsiella pneumoniae* operon induce autophagy

Piscotta et al., 2021, Cell Reports *36*, 109746
September 21, 2021 © 2021 The Authors.
https://doi.org/10.1016/j.celrep.2021.109746

CellPress

Cell Reports

CellPress
OPEN ACCESS

Article

Multiplexed functional metagenomic analysis of the infant microbiome identifies effectors of NF-κB, autophagy, and cellular redox state

Frank J. Piscotta,[1,2] Shawn T. Whitfield,[1,2] Toshiki G. Nakashige,[1,2] Andreia B. Estrela,[1] Thahmina Ali,[1] and Sean F. Brady[1,3,*]
[1]Laboratory of Genetically Encoded Small Molecules, The Rockefeller University, New York, NY 10065, USA
[2]These authors contributed equally
[3]Lead contact
*Correspondence: sbrady@rockefeller.edu
https://doi.org/10.1016/j.celrep.2021.109746

SUMMARY

The human microbiota plays a critical role in host health. Proper development of the infant microbiome is particularly important. Its dysbiosis leads to both short-term health issues and long-term disorders lasting into adulthood. A central way in which the microbiome interacts with the host is through the production of effector molecules, such as proteins and small molecules. Here, a metagenomic library constructed from 14 infant stool microbiomes is analyzed for the production of effectors that modulate three distinct host pathways: immune response (nuclear factor κB [NF-κB] activation), autophagy (LC3-B puncta formation), and redox potential (NADH:NAD ratio). We identify microbiome-encoded bioactive metabolites, including commendamide and hydrogen sulfide and their associated biosynthetic genes, as well as a previously uncharacterized autophagy-inducing operon from *Klebsiella* spp. This work extends our understanding of microbial effector molecules that are known to influence host pathways. Parallel functional screening of metagenomic libraries can be easily expanded to investigate additional host processes.

INTRODUCTION

Mounting evidence suggests that proper development of an infant's gut microbiome is critical to the health of the developing child. Maternal stress, mode of delivery (vaginal versus Cesarian), and feeding patterns (breastfeeding versus formula) are several factors that can cause dysbioses of the infant's gut microbiome, which is often associated with disease in the infant host (Arrieta et al., 2014; Yang et al., 2016; Mohammadkhah et al., 2018). Altered gut microbiome compositions relative to a "healthy" infant are correlated with obesity (reduced *Bacteroidetes* spp.) (Turnbaugh et al., 2006; Zuo et al., 2011), inflammatory bowel disease (overall reduction of bacterial diversity) (Alam et al., 2020; Clooney et al., 2021), and necrotizing enterocolitis (overgrowth of Proteobacteria) (Olm et al., 2019; Lindberg et al., 2020). Critically, adverse development of the infant gut microbiome is thought to also affect health later in life. For example, numerous studies have linked increases in Clostridia and decreases in Bifidobacteria in the first two years of infancy with the development of allergies such as ectopic dermatitis later in life (Reddel et al., 2019; Ismail et al., 2016). The interplay between the gut microbiome and host neurodevelopment (the gut-brain axis) also appears to stretch back into infancy. A recent study in mice has shown that increased prenatal maternal stress can result in long-lasting changes in the infant microbiome associated with anxiety-like behaviors later in life (Gur et al., 2019). Similarly, reduced alpha diversity in the gut microbiomes of one-year-old infants has been linked to changes in cognitive abilities one year later (Carlson et al., 2018). The far-reaching influence of the infant microbiome, as illustrated by these studies, makes it of particular interest among microbial communities.

Studies linking changes in microbial diversity to disease are useful for describing the role of the microbiome in infant development, but a full understanding of the underlying interactions requires identifying and deciphering the mechanisms by which the microbiota signals its host. A major mode of communication between microbe and host is bacterially produced effector molecules (i.e., proteins and small molecules). Both culture-dependent and -independent approaches have been developed to probe these types of interactions and identify bacterially produced effectors. Culture-dependent screening approaches rely on growing commensal bacterial strains and then testing them for the production of bioactive molecules. This allows for direct identification of effector molecules from their microbial producers and has been used to identify numerous human microbiome-derived molecules that modulate host-microbiome interactions, including short-chain fatty acids (Morrison and Preston, 2016; Miller and Wolin, 1996), indoles (Bansal et al., 2010; Chimerel et al., 2014), and secondary bile acids (Kitahara et al., 2001; Ridlon et al., 2006). This approach is limited by difficulties

This is an open access article under the CC BY license (http://creativecommons.org/licenses/by/4.0/).

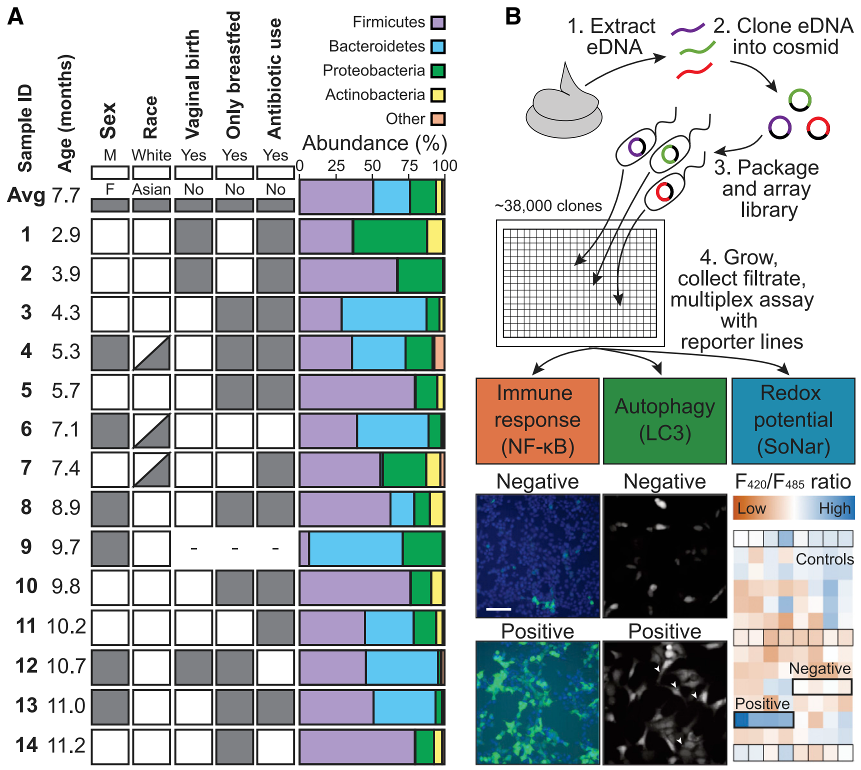

Figure 1. Schematic of functional metagenomics workflow and assays

(A) Demographic information for infants from whom stool samples were collected (dashes indicate where no data were collected) and bacterial composition of infant stool samples as assessed by 16S sequencing.

(B) Genomic DNA from infant stool samples was extracted and cloned into a cosmid library. This library was screened with a diverse set of reporters examining immune response (NF-κB), autophagy (LC3), and redox potential (SoNar) in live-cell-based assays. Positives are as follows: GFP-positive cells for NF-κB, GFP-LC3 puncta accumulation (puncta indicated by white arrows) for LC3, and high or low F_{420}/F_{485} ratio compared with controls (indicated by square boxes highlighted in black) for SoNar. Scale bar, 100 μm. See also Table S1.

associated with culturing roughly 30%–60% of human microbiome-associated bacteria *in vitro* (Lagkouvardos et al., 2017; Walker et al., 2014). Culture-independent effector screening circumvents this limitation by cloning DNA directly from environmental samples followed by screening for activities produced by the resulting metagenomic clones. While this approach has its own limitations, including difficulties associated with heterologous expression of some effector genes in model bacterial hosts, it provides an orthogonal method for identifying microbial effectors that might otherwise be overlooked in culture-dependent studies. Another key advantage of functional metagenomic screening methods is that it couples each hit to a small fragment of cloned DNA, making it possible to not only identify effector molecules but also to readily identify the genes that encode these molecules. Our lab has previously leveraged a culture-independent approach to identify a number of nuclear factor κB (NF-κB)-activating effector genes from adult stool microbiomes (Cohen et al., 2015).

Here, we expanded our culture-independent discovery efforts by screening an infant-derived metagenomic library using a multiplexed assay strategy to probe a suite of cellular processes relevant to the developing infant. We constructed a metagenomic library representative of stool from 14 healthy Western infants and screened this library for bacterial effectors of host immunity (NF-κB signaling), autophagy (LC3-B accumulation), and cellular metabolism (NADH:NAD ratio). This led to our discovery of a number of infant microbiome-derived effector genes that influence each of these pathways. These effector genes encode a diverse set of proteins that are predicted to signal to the host in multiple different ways, including through the production of bioactive small molecules such as commendamide, which induces NF-κB signaling, and hydrogen sulfide, which alters cellular redox potential, and possibly through direct action of the proteins themselves. These effectors also include an uncharacterized six-gene operon found in Gammaproteobacteria, particularly in diverse *Klebsiella* spp., that is predicted to encode components of a type I secretion system, a peptidase, and a glycosyltransferase that confers autophagy activity to *Escherichia coli*. Although this study does not directly link effector molecules from the infant stool microbiome to pathophysiology or host development, it does add to the growing list of microbial products that are known to affect host pathways *in vitro* and provides testable hypotheses for exploring the role the microbiome plays in its complex relationship with its host. In general, the application of multiplexed screening strategies to functional metagenomic discovery efforts provides an opportunity to identify a wider array of bacterial effectors, which should help identify unique mechanisms through which the microbiome influences its host.

RESULTS

Construction of an infant stool microbiome library

To create an infant gut microbiome metagenomic library, we collected fecal samples from healthy 3- to 12-month-old infants in New York City. Samples came from white or Asian infants of both sexes, the majority of which were born via vaginal delivery and included both exclusively breastfed and formula-fed individuals (Figure 1A). Stool was collected from diapers, and microbial DNA was extracted using techniques previously established by our lab (Cohen et al., 2015; Brady, 2007). 16S rDNA sequences from individual metagenomic samples were amplified and sequenced to determine each microbiome's taxonomic composition (Figure 1A; Table S1).

In general, samples were dominated by the Bacteroidetes and Firmicutes phyla, which is typical of most child and adult microbiome compositions in the Western world (Qin et al., 2010; Eckburg et al., 2005), although individual infant stool metagenomes showed significant phyla-level variation in abundance. To focus our discovery efforts on the most common effector genes, DNA from all subjects was pooled, and the pooled sample was then used to construct a metagenomic library that is representative of the microbiome of 3- to 12-month-old infants. Metagenomic DNA was ligated into the pJWC1 cosmid vector (Craig et al., 2009) and introduced into *E. coli* EC100 using lambda phage. Colonies were individually arrayed in 384-well plates to produce a library of ~38,000 unique clones (including 42 empty pJWC1 controls per plate), with each clone carrying an ~30 kbp metagenomic DNA fragment. In total, the library contains over 1 Gbp of stool metagenomic DNA. The final arrayed library was stored as individual glycerol stocks in 384-well plates to permit straightforward pin transfer into fermentation plates for use in effector screening studies.

A collection of cell-based assays captures diverse biological responses to effectors encoded by the infant metagenomic library

The stool DNA library was screened to identify responsive clones in assays representing diverse cellular processes (Figure 1B). These included the following: (1) stress/immune response as measured by NF-κB activation, (2) macroautophagy (hereafter autophagy) induction as measured by LC3-B (hereafter LC3) puncta formation, and (3) changes to cellular redox state as measured by the intracellular ratio of NAD^+ to NADH. We chose these assays to examine a range of cellular activities that might be influenced by the infant microbiome. NF-κB activation is a broad cellular response that we have previously investigated in the adult stool microbiome. We were interested in whether the differing composition of the infant stool microbiome would lead to identification of unique NF-κB effectors. Both autophagy and redox state are dynamic cellular functions, the proper balancing of which is important for proper organismal development and whose dysfunctions can have severe physiological consequences. Since NF-κB signaling (Ramakrishnan et al., 2019), autophagy (Clarke and Simon, 2019; Martinez et al., 2013), and altered redox state (Mesquita et al., 2018; Sánchez-Villanueva et al., 2019) influence host immune responses to pathogens or colonizing organisms (Moy and Cherry, 2013), we expected that gut microbes have developed mechanisms to interact with these pathways.

To measure NF-κB response, we used HEK293 cells stably transfected with a reporter plasmid (pGreenFire, Systems Biosciences) containing a GFP-luciferase operon under the control of four NF-κB transcriptional response elements (Cohen et al., 2015). To monitor autophagy induction, we used HEK293 cells stably transfected with a GFP-LC3 reporter plasmid (Kabeya et al., 2000). The LC3 protein participates in the construction of autophagosomal membranes and is the most commonly used marker for autophagy; a GFP-LC3 fusion allows visualization of autophagosome formation as LC3 is recruited onto the membrane and accumulates into puncta. For monitoring redox potential, NCI-H1299 cells were stably transfected with a "SoNar" reporter plasmid that expresses a yellow fluorescent protein (YFP)-T-Rex fusion protein (Zhao et al., 2015). The binding of NAD^+ or NADH to T-Rex differentially affects the fluorescence of this YFP fusion, providing a means of monitoring the ratio of these two key cellular metabolites in cells. The simplicity of these assays allowed us to parallelize our analysis and screen for microbial effectors of a range of host processes using a single fermentation of our metagenomic library.

Parallel screening to assess diverse phenotypic readouts

To screen for the production of effector molecules, the arrayed metagenomic library was pin transferred into 384-well, 0.2 μm filter plates containing Luria Bertani (LB) broth (Figure 1B). After 4 days, cells were removed by filtration to yield sterile spent culture broth for each clone. The three reporter cell lines were separately arrayed into 384-well plates and then treated with these sterile 4-day-old culture broth filtrates. Following an incubation period (24 h for NF-κB and SoNar, 2 h for LC3), the readout of each reporter was measured (GFP fluorescence for NF-κB, puncta enumeration for LC3, F_{420}/F_{485} fluorescence ratio for SoNar). To ensure responses were not the result of extract toxicity, all wells underwent Hoechst staining and propidium iodide counterstaining to determine the number of live and dead cells, respectively. Within each assay, clones were assigned a *Z* score to measure activation of the desired phenotype, with a *Z* score greater than 3 being considered "active" in our initial screen. Of the 38,000 clones we assayed, 499 were NF-κB active (1.3%; Figure 2A), 339 were LC3 active (0.9%; Figure 2C), and 182 were SoNar active (0.5%, Figure 2E) in the primary screen. These clones were re-assayed in quadruplicate against the appropriate cell line to validate their observed activities. In this secondary screen, 86 clones retained NF-κB activity (17.2% of initial hits, top 10 strongest hits shown in Figure 2B). Five clones reproducibly showed increased LC3 activity (1.5% of initial hits; Figure 2D). Of the five candidates that passed initial SoNar validation (S001–S005; Figure 2F), only one clone (S001) showed reproducible activity and was pursued in more detail (0.5% of initial hits). The higher attrition rate in LC3 and SoNar validation may reflect the sensitivity of these assays. NF-κB provides a largely binary, time-stable readout (cell fluorescence versus non-fluorescence) that is easily quantifiable. By contrast, difficulties associated with the reproducible automated identification of GFP-LC3 puncta and the small dynamic range of the SoNar assay make these two assays more susceptible to noise, thereby resulting in higher false positive rates at the initial high-throughput screening stage. Despite the loss of some clones during the validation process, active clones from our metagenomic library were identified for all three assays (Table S2).

NF-κB activators found in the infant stool metagenome

Of the 82 NF-κB active clones we identified, 37 were unique and 45 were from overlapping genomic regions. Dereplication of the overlapping clones (overlaps shown in Figure S1) resulted in 10 unique genomic regions, leading to a total of 47 NF-κB active effector regions (Figure 3A). Twenty-nine effector regions (62%) mapped closely to sequenced bacterial genomes from the

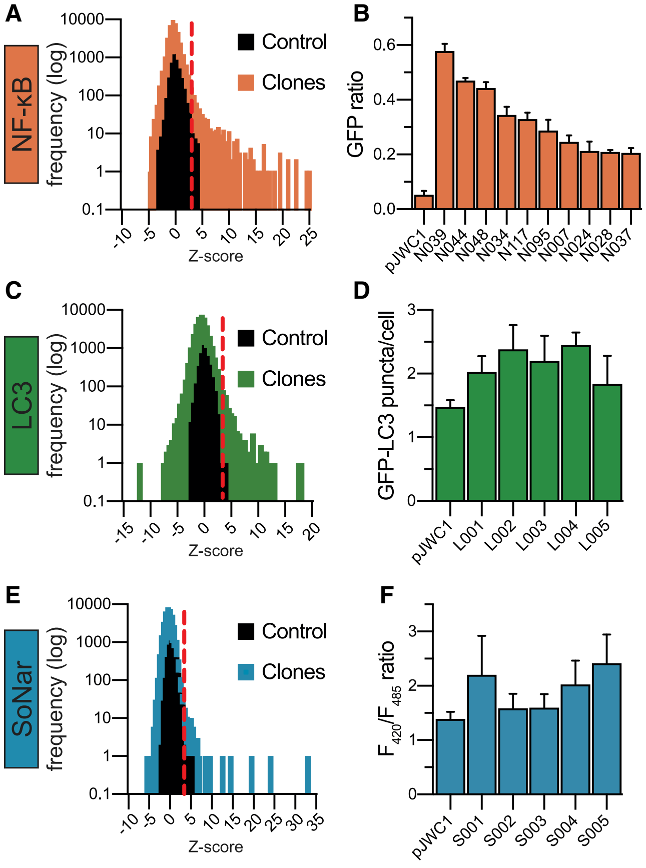

Figure 2. Primary screening and hit validation of the infant metagenomic library assayed using NF-κB, LC3, and SoNar reporter cell lines

(A and B) Distribution of *Z* score frequencies in the NF-κB primary screen and top 10 validated hits from screening.

(C and D) Distribution of *Z* score frequencies in the LC3 primary screen and the five hits that repeatedly validated.

(E and F) Distribution of *Z* score frequencies in the SoNar primary screen and 5 hits from the primary screen that were chosen for validation. Only S001 repeatedly validated in follow-up assays. Clones with a *Z* score greater than 3 (red dotted line) were subsequently tested in validation experiments for activity in all primary screens. Data in (B), (D), and (F) are represented as mean ± SD, n = 4. See also Table S2.

Bacteroidetes, Firmicutes, and Proteobacteria phyla (Figure 3B). The remaining 18 NF-κB active regions (38%) could not be aligned closely to a region from any sequenced bacterium (no matches with >70% coverage, >90% identity). Sequenced bacteria with genomic regions most closely related to these metagenomic sequences that confer NF-κB activity are shown in Table S2. Interestingly, two of the most common effector regions we identified do not map back to any sequenced commensal bacteria (Figure 3C). Even with the extensive sequencing of the human microbiome that has occurred over the past decade, we have identified a number of NF-κB active effector regions that do not appear to have arisen from any sequenced bacteria, illustrating a key advantage of using a culture-independent metagenomic approach for studying the microbiota.

Twenty-one (45%) of the NF-κB active effector regions we identified contain an effector gene seen previously in either an adult stool metagenomic library or a multi-genomic library constructed from common commensal bacteria (Cohen et al., 2015; Estrela et al., 2019). Here, we elected to look in more detail at the most potent common effector regions (i.e., regions in multiple clones) identified in our screen (Figure 3C; Table S2). Among these, there were a number of known effector genes as well as clones that did not encode an obvious previously reported NF-κB effector. Known effector genes include predicted *N*-acyl synthase (NAS), guanosine triphosphate (GTP) pyrophosphokinase, and peptidase p60 genes (Figure 3C) (Estrela et al., 2019; Cohen et al., 2015). In instances where there was no obvious NF-κB effector gene, candidate effector genes could be localized to a small genomic fragment found in all overlapping clones (Figure 3D; Figure S1). We chose the top three strongest NF-κB-activating uncharacterized common effector regions (determined as an average of the NF-κB activation of the group) for follow-up investigation using transposon mutagenesis of two independent cosmid clones associated with each common region (Figure 3D; Figure S2). For each effector region, transposons that knocked out NF-κB activity reproducibly fell into a single gene. These predicted NF-κB effector genes encoded a GLUG-domain-containing protein, a D-glycero-β-D-manno-heptose-7-phosphate kinase, and a MATE (multi-antimicrobial extrusion protein) family efflux transporter (Figure 3D). D-Glycero-β-D-manno-heptose-7-phosphate kinases are involved in lipopolysaccharide (LPS) biosynthesis, for which intermediate products are increasingly being found to activate NF-κB signaling (Gaudet et al., 2015; Pfannkuch et al., 2019; Zimmermann et al., 2017). The cosmid vector used in this study, pJWC1, functions in a broad range of proteobacterial hosts (Craig et al., 2010). To confirm that the MATE transporter was not inducing an NF-κB response as a result of the transport of *E. coli*-specific molecules, we shuttled N044 into three additional hosts: *Caulobacter vibroides* C15, *Agrobacterium tumefaciens* LBA4404, and *Burkholderia graminis* C4D1M. In the case of *A. tumefaciens* LBA4404, we observed that supernatants from cultures transformed with N044 induced the NF-κB reporter cells (Figure S3), indicating that the MATE efflux transporter activity was not specific to *E. coli*. Whether the other strains failed to induce NF-κB activity because they don't express the transporter from this clone or because they don't produce the secreted effector molecule remains to be determined. To the best of our knowledge, roles for GLUG-domain-containing proteins and MATE family efflux transporters in NF-κB activation have not been described, suggesting that these are as-yet uncharacterized pathways through which bacteria can modulate host immune response through NF-κB signaling. Further studies are required to elucidate whether these proteins are directly imparting their bioactivities or whether they are the result of small molecules they produce.

One of the most common effector gene families we identified was the *N*-acetyltransferase family, which was found in 12 NF-κB active clones: ten associated with common genomic regions (Figure 3C) and two from singleton clones (Table S2). In functional metagenomic analyses of the adult stool microbiome, we identified a NAS (Cbeg12) that produces commendamide, an *N*-acylated glycine that agonizes the G-protein-coupled

CellPress
OPEN ACCESS

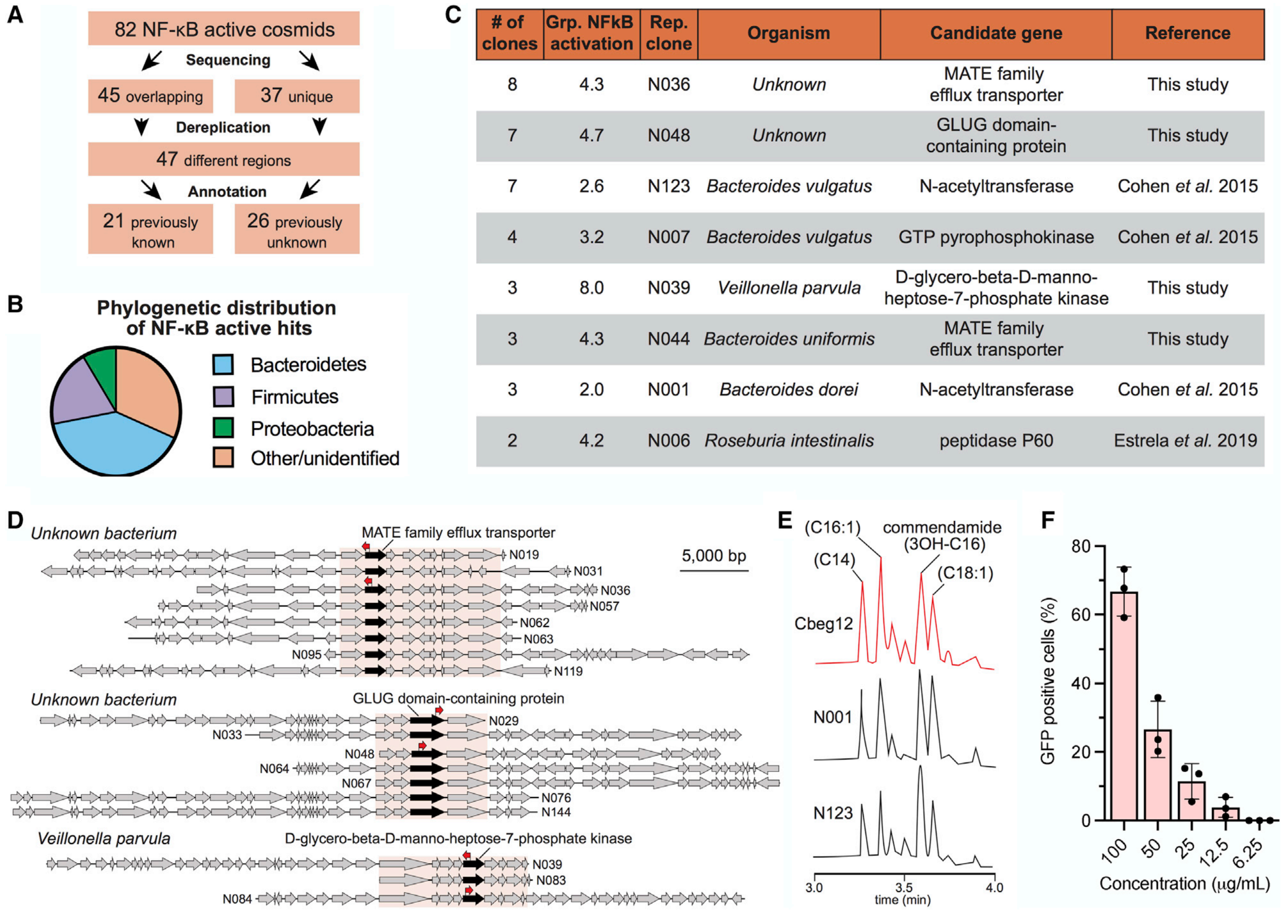

# of clones	Grp. NFkB activation	Rep. clone	Organism	Candidate gene	Reference
8	4.3	N036	*Unknown*	MATE family efflux transporter	This study
7	4.7	N048	*Unknown*	GLUG domain-containing protein	This study
7	2.6	N123	*Bacteroides vulgatus*	N-acetyltransferase	Cohen *et al.* 2015
4	3.2	N007	*Bacteroides vulgatus*	GTP pyrophosphokinase	Cohen *et al.* 2015
3	8.0	N039	*Veillonella parvula*	D-glycero-beta-D-manno-heptose-7-phosphate kinase	This study
3	4.3	N044	*Bacteroides uniformis*	MATE family efflux transporter	This study
3	2.0	N001	*Bacteroides dorei*	N-acetyltransferase	Cohen *et al.* 2015
2	4.2	N006	*Roseburia intestinalis*	peptidase P60	Estrela *et al.* 2019

Figure 3. Infant microbiome-derived NF-κB activators

(A) Of 82 NF-κB active clones, 45 contained cosmid vectors with genomic regions bearing significant overlap with other clones and 37 contained cosmid vectors with unique genomic regions. Altogether, 21 regions contained genes previously identified as NF-κB activators and 26 contained uncharacterized effectors.

(B) Taxonomic distribution of sequenced NF-κB active clones at the phylum level.

(C) Breakdown of overlapping groups for which candidate NF-κB active genes were previously identified or identified in this study.

(D) For clones with no previously characterized NF-κB activators, transposon mutagenesis was performed on two representative clones from each genomic region (highlighted in pale orange). Transposon insertions causing loss of NF-κB activation (red arrow) were used to identify candidate effector genes.

(E) Representative LC-MS traces of culture broths from N001 and N123 (representative *N*-acetyltransferase clones) compared with the known commendamide producer Cbeg12. Peaks diagnostic of commendamide and related products appear in all three culture broths.

(F) Activation of NF-κB reporter cells by commendamide. Data are represented as mean ± SD, n = 3. See also Figures S2, S3, and S4 and Table S3.

receptor (GPCR) G2A. G2A has been implicated in autoimmune disease and atherosclerosis (Cohen et al., 2015). Our subsequent analysis of NASs encoded by the microbiota found that that they acylate a variety of primary and secondary amines to yield long-chain *N*-acyl products that agonize diverse GPCRs (Cohen et al., 2015, 2017). Two of the most common NF-κB effector regions we identified from the infant stool microbiome contained predicted NAS genes. We examined the lipid content of *E. coli* transformed with clones containing each of these NAS genes, or cbeg12, to determine whether the infant microbiome encodes the same *N*-acyl lipids as the adult microbiome. Liquid chromatography-mass spectrometry (LC-MS) analysis of culture broth extracts from these clones (Figure S4) showed that they produced commendamide as well as related structures containing fatty acids of different length (Figure 3E) that are identical to those found in the adult microbiome (cbeg12). Commendamide induced NF-κB reporter cell fluorescence in a dose-dependent manner (Figure 3F), confirming the NF-κB activity of this metabolite. These results indicate that microbiome-derived NF-κB active small molecules such as commendamide are not merely transiently presently, but are present throughout human development from infancy to adulthood.

The occurrence of proteins encoded by these NF-κB active genes was examined in metagenomic samples collected from 286 patients and deposited in the Human Microbiome Project (HMP) database. For each gene family, the protein encoded by a representative clone was BLAST searched against each patient metagenome and highly similar proteins (>90% identity, >70% coverage) were identified (Table S3). Of the 11 representative clones examined, the predicted D-glycero-β-D-manno-heptose-7-phosphate kinase (N039) was the most frequently seen protein, occurring in 40 of the 286 patient samples;

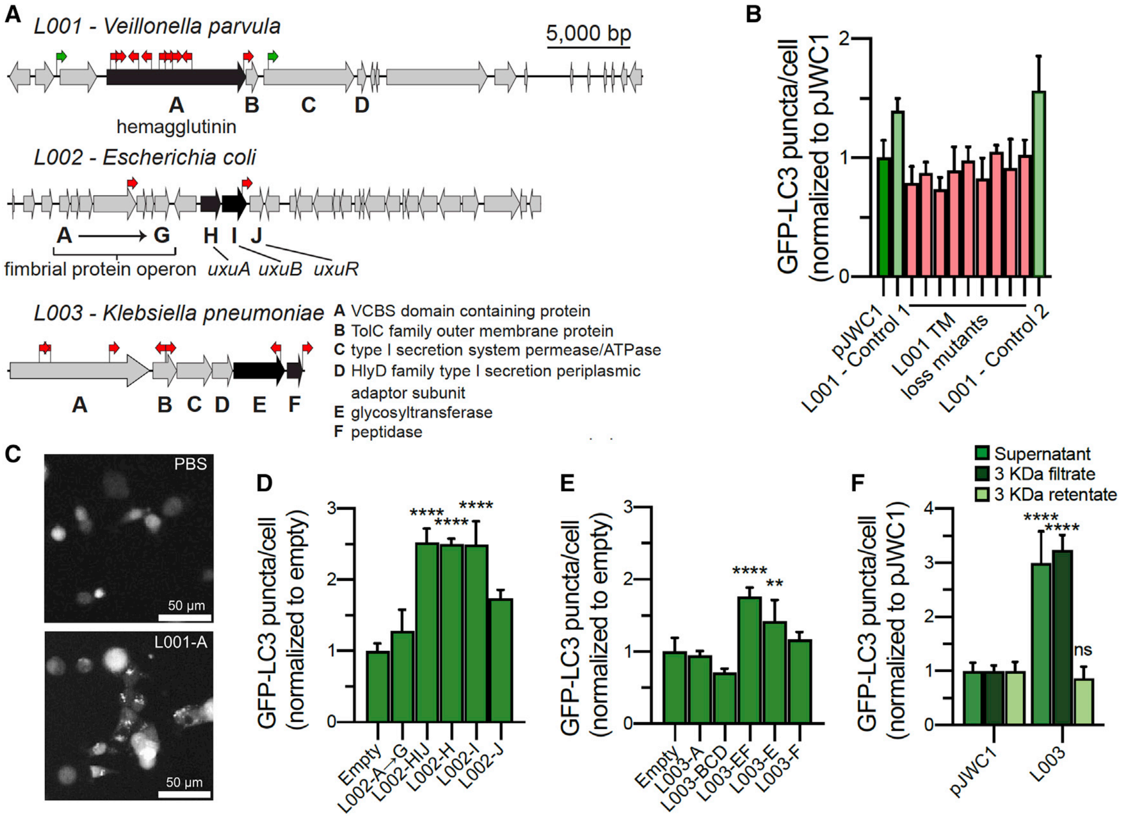

Figure 4. Autophagy-inducing clones from the infant microbiome metagenomic library

(A) Transposon mutagenesis of three LC3-active clones to identify effector genes. A red arrow indicates the position of an inserted transposon associated with loss of clone activity. Black highlighting indicates candidate autophagy-inducing genes. The hemagglutinin gene from *V. parvula* and *uxuAB* gene cluster from *E. coli* are known to affect autophagic pathways.

(B) Transposon mutagenesis of L001. Controls and mutants are given in the same order (left to right) as arrows in (A). Data are normalized to empty vector controls and represented as mean ± SD, n = 4.

(C) Purified L001-A induces autophagy in LC3 reporter cells. LC3 cells were treated with either PBS (top) or L001-A (0.5 mg/mL; bottom) for 2 h and monitored for LC3 accumulation.

(D and E) Subcloning of candidate gene regions to identify those responsible for autophagy induction in the LC3 reporter cell line. Data are normalized to empty vector controls and represented as mean ± SD, n = 4. Ordinary one-way ANOVA with Dunnett's multiple comparisons test: **$p \leq 0.01$; ****$p < 0.0001$.

(F) The L003 cosmid supernatant retains activity when passed through a 3 kDa molecular weight cutoff membrane. Ordinary one-way ANOVA with Dunnett's multiple comparisons test: ns, no significance; ****$p < 0.0001$. Data are normalized to empty vector controls and represented as mean ± SD, n = 4. Scale bar, 50 μm. See also Figure S2 and Table S3.

conversely, the GLUG-domain-containing protein (N029) was only identified in a single patient. Of the 286 patient metagenomes examined, 163 (57%) contained at least one NF-κB active gene, demonstrating that although each individual effector gene may have low abundance, on the whole these genes are found in a majority of patients.

Effectors of autophagy from the infant stool microbiome

Of the five clones active in the LC3 autophagy assay, which we termed L001 through L005, sequencing revealed three unique genomic regions. One region was shared by L001 and L004, one was shared by L002 and L005, and one was unique to L003. Based on high sequence identity to sequenced bacterial genomes (>90%), the metagenomic inserts captured in these clones are predicted to arise from *Veillonella parvula* (L001/L004), *E. coli* (L002/L005), and *Klebsiella pneumoniae* (L003). We used transposon mutagenesis to identify the gene(s) responsible for the autophagy-inducing activity conferred by each clone.

Transposon mutagenesis of clone L001 (Figures 4A and 4B) showed that all of the inactivating transposons we identified fell in the first gene (*L001-A*) of a four-gene operon we termed *L001-ABCD*. *L001-A* is annotated as a YadA-like family/hemagglutinin protein. Hemagglutinins are known autophagy modulators that can either inhibit (*Mycobacterium tuberculosis* hemagglutinin) or promote (viral hemagglutinin) autophagy (Zheng et al., 2017; Zhirnov and Klenk, 2013; Pan et al., 2014). Although *L001-C* is also predicted to encode a hemagglutinin, it differs in that L001-A contains two EmaA (collagen-binding adhesion autotransporter) domains, while L001-C only contains one. This difference in domain architecture is presumed to be important for the autophagy activity of L001-A; however, in general, it is

difficult to distinguish between autophagy-inducing and non-inducing hemagglutinins by sequence identity or domain organization. For example, among the 18 known influenza A virus hemagglutinin subtypes only a subset has been found experimentally to directly stimulate autophagosome production (Zhirnov and Klenk, 2013). To test L001-A LC3 activity, the encoding gene was cloned into pET28c for expression in *E. coli* and the purified recombinant protein was applied to LC3 reporter cells. As suggested by the transposon mutagenesis study, addition of L001-A activated LC3 response (Figure 4C). From among the many hemagglutinins present in the human microbiome, our functional analysis of the infant stool microbiome allowed us to assign autophagy activity to the L001-A-like subclass of hemagglutinins. Based on sequence identity (>90% identity) and similar domain organization, L001-A-like hemagglutinins are found only in *V. parvula* and *Veillonella dispar* among the 15 classified *Veillonella* species that have been isolated from the microbiota. In patient samples deposited in the HMP, L001-A-like hemagglutinin proteins (>90% identity, >70% coverage) were more widespread than products of other phenotypically active genes, occurring in 99 of the 286 patient metagenomes.

Transposon mutagenesis of clone L002 narrowed the candidate effector genes to two predicted operons, one with 7 genes (*L002-ABCDEFG*) and the other with 3 genes (*L002-HIJ*) (Figure 4A; Figure S2). We have previously observed that some pJWC1-based cosmids can exhibit instability resulting in false transposon knockouts. To identify the specific autophagy-inducing genes in L002, we subcloned the genes from these operons. Candidate genes were individually cloned into pET28c for heterologous expression in *E. coli*, with the exception of *L002-ABCDEFG*, which was cloned as an operon. Filtered supernatants from cultures expressing these individual genes or operons were assayed against the LC3 reporter cell line. Supernatants from cells expressing the *L002-ABCDEFG* operon did not induce autophagy, while supernatants from cells expressing *L002-H*, *L002-I*, and *L002-J* individually, as well as the full *L002-HIJ* operon, all significantly increased GFP-LC3 puncta per cell above a negative control (Figure 4D). The largest increase was observed in *L002-H* and *L002-I* supernatants, and these two genes were examined further. The predicted L002-H and L002-I proteins are 100% identical to *E. coli* K-12 mannonate dehydrogenase, UxuA, and fructuronate reductase, UxuB, two enzymes that are part of the pentose-glucuronate interconversion pathway. Similarly, L002-J is identical to the Uxu operon regulator in *E. coli*. While this pathway itself has not been implicated in autophagic response, the substrate of UxuA, 2-dehydro-3-deoxy-D-gluconic acid, is used in the pentose phosphate pathway, of which the enzyme ribose-5-phosphate isomerase has been shown to inhibit autophagic response (Heintze et al., 2016). Given this link to autophagy and the interconnected nature of the pentose-glucuronate and pentose-phosphate pathways, it is likely that a change in the availability of the 2-dehydro-3-deoxy-D-gluconic acid intermediate could significantly affect autophagic activity in the host; however, the possibility remains that these proteins may be imparting their autophagy-inducing activity directly. *L002-H* and *L002-I* were subcloned into pET28c, and cultures of *E. coli* expressing these constructs were examined by high-resolution MS and SDS-PAGE for gene-specific small molecules and proteins, respectively. No unique molecules were observed compared with an empty pET28c control culture. We also assayed glucuronic and galacturonic acid from the pentose-glucoronate pathway, but neither showed LC3 activity. Determining the exact product of the pentose-phosphate pathway that induces LC3 activity will require additional investigation.

The insert in clone L003 was shorter than expected (17.9 kbp) because this clone contained two copies of the pJWC1 cosmid vector. The captured metagenomic fragment contains a single six-gene operon (*L003-ABCDEF*). Transposons that knocked out autophagy activity conferred by this clone fell throughout this entire operon (Figure 4A; Figure S2). To identify the specific effector gene(s) in this operon, we subcloned subsets of the L003 genes into pET28c and assayed for their ability to confer autophagy activity to *E. coli*. Culture broth from the fermentation of *E. coli* transformed with the plasmid expressing *L003-E* alone induced autophagy in reporter cells (Figure 4E). Interestingly, the autophagy response was stronger when *L003-E* and *L003-F* were subcloned together, which suggests an additive effect that may arise from one gene product creating a substrate used by the other. *L003-E* and *L003-F* are predicted to encode a glycosyltransferase and peptidase, respectively. The remaining genes in the L003 operon are predicted to encode a VCBS-domain protein (L003-A) as well as parts of a type I secretion system (L003-BCD).

Unlike for clones L001 and L002, there is no obvious link between the proteins encoded by the L003 operon and known autophagy-inducing pathways. To investigate the distribution of the L003 operon, we searched sequenced bacterial genomes for close homologs (>90% coverage, >40% identity) of L003-E. This search revealed that homologs were distributed uniquely among Gammaproteobacteria, appearing in reference genomes belonging to 27 species that were mainly distributed in the Erwiniaceae and Enterobacteriaceae families. Within the *Klebsiella* genus, L003-E is found in 7 of the 19 classified *Klebsiella* species. The full L003 operon was present in in all 7 of these species as well as 9 additional species that possess an *L003-E* homolog, suggesting that the genes in the L003 operon likely function together in their native bacteria.

To elucidate the mechanism by which the L003 operon confers to *E. coli* the ability to induce autophagy, culture broth from *E. coli* bearing the L003 cosmid was passed through a 3 kDa molecular weight cutoff membrane. When the flowthrough and washed retentate were assayed for LC3 activity, activity was found in the flowthrough, suggesting that the effector is a secreted small molecule (Figure 4F). Supernatants from *E. coli* expressing *L003-EF* or an empty pET28c vector were analyzed by untargeted LC coupled to high-resolution MS (LC-HRMS), but to date we have not been able to identify statistically significant differences in small molecules between these cultures. While our data suggest that the autophagy-inducing activity conferred to *E. coli* by L003 is the result of a small molecule, further work is necessary to determine the exact mechanism by which L003 induces an autophagic response in eukaryotic cells. *K. pneumoniae*, the predicted source of the L003 operon, has been shown to induce autophagy both in *in vitro* cell cultures (Shi et al.,

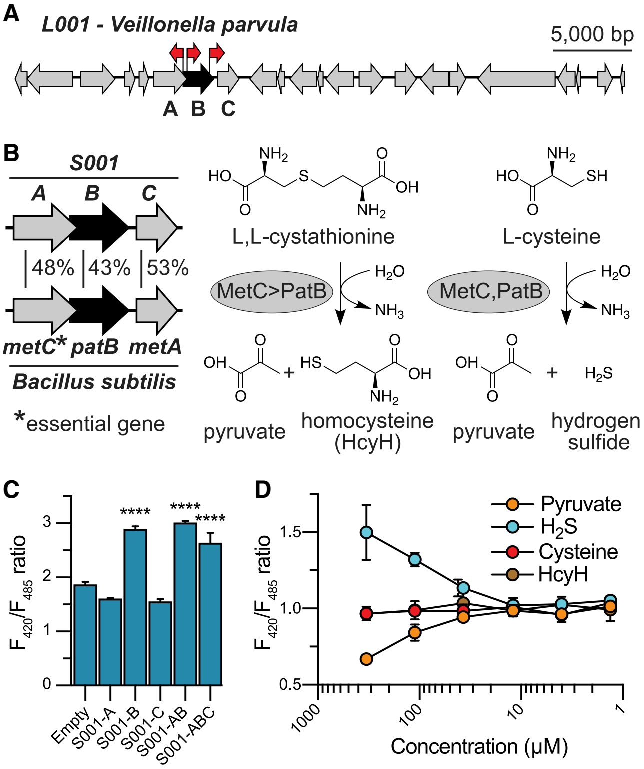

Figure 5. SoNar-active clone identified from the infant metagenomic library

(A) Transposon mutagenesis of clone S001 identified three candidate genes for inducing reporter cell response. A red arrow indicates the position of an inserted transposon associated with loss of clone activity. Black highlighting indicates candidate autophagy-inducing genes.

(B) S001-A and S001-B are homologous to cystathionine β-lyase proteins MetC and PatB, respectively. Only MetC is necessary for cell growth, but PatB can partially complement a loss of MetC. Substrate specificity for the MetC and PatB β-lyase families differs for the reactions they catalyze.

(C) Subcloning of *S001-ABC* genes shows that *S001-B* is necessary and sufficient for inducing cellular response. Data are represented as mean ± SD, n = 4. Ordinary one-way ANOVA with Dunnett's multiple comparisons test: ****p < 0.0001.

(D) SoNar assay of metabolic products of PatB shows that hydrogen sulfide alone increases the F_{420}/F_{485} ratio. Data are represented as mean ± SD, n = 4. See also Figure S2 and Table S3.

2020) as well as in an *in vivo Caenorhabditis elegans* model (Kamaladevi and Balamurugan, 2017). This study suggests that the L003 operon should be explored for a potential role in this phenotype.

SoNar activity from the infant stool microbiome

Screening for microbiome-derived modulators of cellular redox potential yielded a single reproducibly active clone (S001). Based on overall nucleotide sequence identity, the S001 insert could not be traced back to a sequenced bacterium. The closest sequenced genomic region we identified was from *V. parvula* (91% sequence identity but only 28% coverage). Transposon mutagenesis suggested that the active gene in S001 fell in a group of three genes (*S001-ABC*; Figure 5A; Figure S2). BLASTp queries of these proteins revealed similarity to enzymes associated with the methionine biosynthetic pathway (Figure 5B). S001-A is homologous to the *Bacillus subtilis* MetC enzyme (48% identity to MetC), a cystathionine β-lyase that cleaves L,L-cystathionine to generate homocysteine, the precursor to methionine. Like S001-A, S001-B is also a cystathionine β-lyase. However, S001-B is more similar to proteins from the *B. subtilis* PatB β-lyase family (43% identity) than the MetC β-lyase family. While PatB lyases catalyze the same reactions as the MetC family, they display different substrate specificity, and unlike MetC family lyases, they are not essential for cell growth (Auger et al., 2005). The physiological role of the PatB lyase family is still not well understood. The third protein encoded by this operon, S001-C, bears homology to the *B. subtilis* MetA family of enzymes, which encode homoserine acetyltransferases (53% identity). MetA is also critical to methionine biosynthesis but operates in a different part of the biosynthetic pathway than cystathionine β-lyases.

Each individual gene as well as *S001-AB* and *S001-ABC* was cloned into pET28c for heterologous expression in *E. coli*. Supernatants from cells expressing these genes were filtered and assayed for activity using the SoNar cell line. An increase in SoNar response was observed in reporter cells treated with either *S001-ABC*, *S001-AB*, or *S001-B* supernatants (Figure 5C), suggesting that the cystathionine β-lyase S001-B is responsible for the altered cellular redox potential induced by cultures of clone S001. Unlike what we saw with *L003-EF,* there was no additive effect observed in the *S001-AB* and *S001-ABC* supernatants. Supernatants from cultures of *E. coli* transformed with either the *S001-B* expression vector or an empty pET28c control were analyzed by LC-HRMS, and no differences in composition of small molecules were detected. Some of the products of the PatB enzyme are very low molecular weight (pyruvate) and volatile (hydrogen sulfide), making them difficult to detect in culture media using untargeted MS methods. To determine whether these products could directly drive the response seen in the SoNar assay, we dosed SoNar reporter cells with cysteine, homocysteine, pyruvate, and hydrogen sulfide. Hydrogen sulfide significantly increased the F_{420}/F_{485} ratio of SoNar reporter cells (Figure 5D), suggesting that its production by S001-B is likely causing the observed changes in cellular redox state. Indeed, it has been shown that both endogenous and exogenous hydrogen sulfide can affect the redox potential of eukaryotic cells (Stein and Bailey, 2013); furthermore, hydrogen sulfide concentrations comparable with those driving the SoNar response (high micromolar to low millimolar) have been observed in the human intestinal lumen (Magee et al., 2000).

Production of hydrogen sulfide in the gut is known to occur through two major pathways: the well-characterized sulfate reduction pathway (Dordević et al., 2020) and cysteine fermentation (Blachier et al., 2019). Although dedicated cysteine desulfhydrase enzymes exist to perform cysteine fermentation in the gut microbiome (e.g., in *Clostridium* spp.) (Washio et al., 2014), the results here suggest that the SoNar-active PatB homolog S001-B from an uncharacterized microbiome-derived bacterium may also play a role in the generation of hydrogen sulfide. Proteins that were highly similar (>90% identity, >70% coverage) to SoNar-active S001-B were found in 26 of the 286 patient

metagenomes we examined. The identification of a cysteine fermentation enzyme that can alter energy metabolism in host cells through hydrogen sulfide production is of particular interest in the infant microbiome, as pediatric patients with intestinal disorders such as Crohn's disease has been linked to an abundance of cysteine-fermenting bacteria (Mottawea et al., 2016).

DISCUSSION

The functional metagenomic approach applied here to an infant stool microbiome-derived library was used to perform a multiplexed screen for effectors of NF-κB activation, autophagy induction, and cellular redox potential modulation. The effectors we identified include small molecules such as commendamide and hydrogen sulfide that induce NF-κB signaling and alter cellular redox potential, respectively. We also identified a six-gene operon found within a number of *Klebsiella* spp. that is capable of inducing autophagy through a glycosyltransferase-produced low-molecular weight metabolite. While we cannot link these effectors directly to changes in disease progression or development, functional metagenomics nonetheless allowed us to identify genes and their associated products that can induce biologically relevant phenotypes. These findings also illustrate how functional metagenomics can be used to probe diverse cellular pathways. This multiplexed screening approach can be easily extended to other physiologically relevant bioassays (e.g., tumorigenesis and kinase engagement). Discovery of these bacterial effectors of host physiology will allow us to develop testable hypotheses for future studies characterizing the roles of these molecules within the infant microbiome.

STAR★METHODS

Detailed methods are provided in the online version of this paper and include the following:

- KEY RESOURCES TABLE
- RESOURCE AVAILABILITY
  - Lead contact
  - Materials availability
  - Data and code availability
- EXPERIMENTAL MODEL AND SUBJECT DETAILS
  - Cell lines and culture conditions
- METHOD DETAILS
  - Metagenomic library construction
  - 16S sequencing and analysis
  - Generation and high-throughput screening of sterile spent culture broth filtrates
  - Sequence annotation of inserts from bioactive clones and identification of effector genes by transposon mutagenesis
  - Identification of effector genes in patient metagenomes
  - Broad proteobacterial expression of MATE-family transporters
  - LC-MS analysis of N-acetyltransferase-containing clones
  - NF-kB activation by pure commendamide
  - Subcloning and inducible expression of effector genes
  - LC3 activation by L001-A
- QUANTIFICATION AND STATISTICAL ANALYSIS

SUPPLEMENTAL INFORMATION

Supplemental information can be found online at https://doi.org/10.1016/j.celrep.2021.109746.

ACKNOWLEDGMENTS

We thank the Rockefeller University High-Throughput Screening and Flow Cytometry Resource centers for expertise and equipment. This work was supported by the Bill and Melinda Gates Foundation grant OPP1168674 and NIH grant 5R01AT009562.

AUTHOR CONTRIBUTIONS

Conceptualization, F.J.P., S.T.W., T.G.N., and S.F.B.; methodology, F.J.P., S.T.W., T.G.N., A.B.E., and S.F.B.; investigation, F.J.P., S.T.W., T.G.N., and A.B.E.; formal analysis, T.A.; writing – original draft, F.J.P., S.T.W., and S.F.B.; writing – review & editing, F.J.P. and S.F.B.; visualization, S.T.W.; supervision, S.F.B.; funding acquisition, S.F.B.

DECLARATION OF INTERESTS

The authors declare no competing interests. S.F.B. is co-founder and academic advisor of Lodo Therapeutics.

Received: December 29, 2020
Revised: July 19, 2021
Accepted: August 31, 2021
Published: September 21, 2021

REFERENCES

Alam, M.T., Amos, G.C.A., Murphy, A.R.J., Murch, S., Wellington, E.M.H., and Arasaradnam, R.P. (2020). Microbial imbalance in inflammatory bowel disease patients at different taxonomic levels. Gut Pathog. *12*, 1.

Almagro Armenteros, J.J., Tsirigos, K.D., Sønderby, C.K., Petersen, T.N., Winther, O., Brunak, S., von Heijne, G., and Nielsen, H. (2019). SignalP 5.0 improves signal peptide predictions using deep neural networks. Nat. Biotechnol. *37*, 420–423.

Arrieta, M.-C., Stiemsma, L.T., Amenyogbe, N., Brown, E.M., and Finlay, B. (2014). The intestinal microbiome in early life: health and disease. Front. Immunol. *5*, 427.

Auger, S., Gomez, M.P., Danchin, A., and Martin-Verstraete, I. (2005). The PatB protein of Bacillus subtilis is a C-S-lyase. Biochimie *87*, 231–238.

Bansal, T., Alaniz, R.C., Wood, T.K., and Jayaraman, A. (2010). The bacterial signal indole increases epithelial-cell tight-junction resistance and attenuates indicators of inflammation. Proc. Natl. Acad. Sci. USA *107*, 228–233.

Blachier, F., Beaumont, M., and Kim, E. (2019). Cysteine-derived hydrogen sulfide and gut health: a matter of endogenous or bacterial origin. Curr. Opin. Clin. Nutr. Metab. Care *22*, 68–75.

Brady, S.F. (2007). Construction of soil environmental DNA cosmid libraries and screening for clones that produce biologically active small molecules. Nat. Protoc. *2*, 1297–1305.

Carlson, A.L., Xia, K., Azcarate-Peril, M.A., Goldman, B.D., Ahn, M., Styner, M.A., Thompson, A.L., Geng, X., Gilmore, J.H., and Knickmeyer, R.C. (2018). Infant Gut Microbiome Associated With Cognitive Development. Biol. Psychiatry *83*, 148–159.

Chimerel, C., Emery, E., Summers, D.K., Keyser, U., Gribble, F.M., and Reimann, F. (2014). Bacterial metabolite indole modulates incretin secretion from intestinal enteroendocrine L cells. Cell Rep. *9*, 1202–1208.

Clarke, A.J., and Simon, A.K. (2019). Autophagy in the renewal, differentiation and homeostasis of immune cells. Nat. Rev. Immunol. *19*, 170–183.

Clooney, A.G., Eckenberger, J., Laserna-Mendieta, E., Sexton, K.A., Bernstein, M.T., Vagianos, K., Sargent, M., Ryan, F.J., Moran, C., Sheehan, D., et al. (2021). Ranking microbiome variance in inflammatory bowel disease: a large longitudinal intercontinental study. Gut *70*, 499–510.

Cohen, L.J., Kang, H.-S., Chu, J., Huang, Y.-H., Gordon, E.A., Reddy, B.V.B., Ternei, M.A., Craig, J.W., and Brady, S.F. (2015). Functional metagenomic discovery of bacterial effectors in the human microbiome and isolation of commendamide, a GPCR G2A/132 agonist. Proc. Natl. Acad. Sci. USA *112*, E4825–E4834.

Cohen, L.J., Esterhazy, D., Kim, S.H., Lemetre, C., Aguilar, R.R., Gordon, E.A., Pickard, A.J., Cross, J.R., Emiliano, A.B., Han, S.M., et al. (2017). Commensal bacteria make GPCR ligands that mimic human signalling molecules. Nature *549*, 48–53.

Craig, J.W., Chang, F.-Y., and Brady, S.F. (2009). Natural products from environmental DNA hosted in Ralstonia metallidurans. ACS Chem. Biol. *4*, 23–28.

Craig, J.W., Chang, F.-Y., Kim, J.H., Obiajulu, S.C., and Brady, S.F. (2010). Expanding small-molecule functional metagenomics through parallel screening of broad-host-range cosmid environmental DNA libraries in diverse proteobacteria. Appl. Environ. Microbiol. *76*, 1633–1641.

Dordević, D., Jančíková, S., Vítězová, M., and Kushkevych, I. (2020). Hydrogen sulfide toxicity in the gut environment: Meta-analysis of sulfate-reducing and lactic acid bacteria in inflammatory processes. J. Adv. Res. *27*, 55–69.

Eckburg, P.B., Bik, E.M., Bernstein, C.N., Purdom, E., Dethlefsen, L., Sargent, M., Gill, S.R., Nelson, K.E., and Relman, D.A. (2005). Diversity of the human intestinal microbial flora. Science *308*, 1635–1638.

Estrela, A.B., Nakashige, T.G., Lemetre, C., Woodworth, I.D., Weisman, J.L., Cohen, L.J., and Brady, S.F. (2019). Functional Multigenomic Screening of Human-Associated Bacteria for NF-κB-Inducing Bioactive Effectors. MBio *10*, e02587-19.

Gaudet, R.G., Sintsova, A., Buckwalter, C.M., Leung, N., Cochrane, A., Li, J., Cox, A.D., Moffat, J., and Gray-Owen, S.D. (2015). INNATE IMMUNITY. Cytosolic detection of the bacterial metabolite HBP activates TIFA-dependent innate immunity. Science *348*, 1251–1255.

Gur, T.L., Palkar, A.V., Rajasekera, T., Allen, J., Niraula, A., Godbout, J., and Bailey, M.T. (2019). Prenatal stress disrupts social behavior, cortical neurobiology and commensal microbes in adult male offspring. Behav. Brain Res. *359*, 886–894.

Heintze, J., Costa, J.R., Weber, M., and Ketteler, R. (2016). Ribose 5-phosphate isomerase inhibits LC3 processing and basal autophagy. Cell. Signal. *28*, 1380–1388.

Ismail, I.H., Boyle, R.J., Licciardi, P.V., Oppedisano, F., Lahtinen, S., Robins-Browne, R.M., and Tang, M.L. (2016). Early gut colonization by Bifidobacterium breve and B. catenulatum differentially modulates eczema risk in children at high risk of developing allergic disease. Pediatr. Allergy Immunol. *27*, 838–846.

Kabeya, Y., Mizushima, N., Ueno, T., Yamamoto, A., Kirisako, T., Noda, T., Kominami, E., Ohsumi, Y., and Yoshimori, T. (2000). LC3, a mammalian homologue of yeast Apg8p, is localized in autophagosome membranes after processing. EMBO J. *19*, 5720–5728.

Kamaladevi, A., and Balamurugan, K. (2017). Global Proteomics Revealed *Klebsiella pneumoniae* Induced Autophagy and Oxidative Stress in *Caenorhabditis elegans* by Inhibiting PI3K/AKT/mTOR Pathway during Infection. Front. Cell. Infect. Microbiol. *7*, 393.

Kitahara, M., Takamine, F., Imamura, T., and Benno, Y. (2001). Clostridium hiranonis sp. nov., a human intestinal bacterium with bile acid 7alpha-dehydroxylating activity. Int. J. Syst. Evol. Microbiol. *51*, 39–44.

Lagkouvardos, I., Overmann, J., and Clavel, T. (2017). Cultured microbes represent a substantial fraction of the human and mouse gut microbiota. Gut Microbes *8*, 493–503.

Lindberg, T.P., Caimano, M.J., Hagadorn, J.I., Bennett, E.M., Maas, K., Brownell, E.A., and Matson, A.P. (2020). Preterm infant gut microbial patterns related to the development of necrotizing enterocolitis. J. Matern. Fetal Neonatal Med. *33*, 349–358.

Lu, J., Breitwieser, F.P., Thielen, P., and Salzberg, S.L. (2017). Bracken: estimating species abundance in metagenomics data. PeerJ Comput. Sci. *3*, e104.

Magee, E.A., Richardson, C.J., Hughes, R., and Cummings, J.H. (2000). Contribution of dietary protein to sulfide production in the large intestine: an in vitro and a controlled feeding study in humans. Am. J. Clin. Nutr. *72*, 1488–1494.

Martinez, J., Verbist, K., Wang, R., and Green, D.R. (2013). The relationship between metabolism and the autophagy machinery during the innate immune response. Cell Metab. *17*, 895–900.

Mesquita, I., Vergnes, B., and Silvestre, R. (2018). Alterations on Cellular Redox States upon Infection and Implications for Host Cell Homeostasis. In Metabolic Interaction in Infection, R. Silvestre and E. Torrado, eds. (Springer International Publishing).

Miller, T.L., and Wolin, M.J. (1996). Pathways of acetate, propionate, and butyrate formation by the human fecal microbial flora. Appl. Environ. Microbiol. *62*, 1589–1592.

Mohammadkhah, A.I., Simpson, E.B., Patterson, S.G., and Ferguson, J.F. (2018). Development of the Gut Microbiome in Children, and Lifetime Implications for Obesity and Cardiometabolic Disease. Children (Basel) *5*, 160.

Morrison, D.J., and Preston, T. (2016). Formation of short chain fatty acids by the gut microbiota and their impact on human metabolism. Gut Microbes *7*, 189–200.

Mottawea, W., Chiang, C.-K., Mühlbauer, M., Starr, A.E., Butcher, J., Abujamel, T., Deeke, S.A., Brandel, A., Zhou, H., Shokralla, S., et al. (2016). Altered intestinal microbiota-host mitochondria crosstalk in new onset Crohn's disease. Nat. Commun. *7*, 13419.

Moy, R.H., and Cherry, S. (2013). Antimicrobial autophagy: a conserved innate immune response in Drosophila. J. Innate Immun. *5*, 444–455.

Olm, M.R., Bhattacharya, N., Crits-Christoph, A., Firek, B.A., Baker, R., Song, Y.S., Morowitz, M.J., and Banfield, J.F. (2019). Necrotizing enterocolitis is preceded by increased gut bacterial replication, *Klebsiella*, and fimbriae-encoding bacteria. Sci. Adv. *5*, eaax5727.

Pan, H., Zhang, Y., Luo, Z., Li, P., Liu, L., Wang, C., Wang, H., Li, H., and Ma, Y. (2014). Autophagy mediates avian influenza H5N1 pseudotyped particle-induced lung inflammation through NF-κB and p38 MAPK signaling pathways. Am. J. Physiol. Lung Cell. Mol. Physiol. *306*, L183–L195.

Pfannkuch, L., Hurwitz, R., Traulsen, J., Sigulla, J., Poeschke, M., Matzner, L., Kosma, P., Schmid, M., and Meyer, T.F. (2019). ADP heptose, a novel pathogen-associated molecular pattern identified in *Helicobacter pylori*. FASEB J. *33*, 9087–9099.

Qin, J., Li, R., Raes, J., Arumugam, M., Burgdorf, K.S., Manichanh, C., Nielsen, T., Pons, N., Levenez, F., Yamada, T., et al. (2010). A human gut microbial gene catalogue established by metagenomic sequencing. Nature *464*, 59–65.

Ramakrishnan, S.K., Zhang, H., Ma, X., Jung, I., Schwartz, A.J., Triner, D., Devenport, S.N., Das, N.K., Xue, X., Zeng, M.Y., et al. (2019). Intestinal non-canonical NFκB signaling shapes the local and systemic immune response. Nat. Commun. *10*, 660.

Reddel, S., Del Chierico, F., Quagliariello, A., Giancristoforo, S., Vernocchi, P., Russo, A., Fiocchi, A., Rossi, P., Putignani, L., and El Hachem, M. (2019). Gut microbiota profile in children affected by atopic dermatitis and evaluation of intestinal persistence of a probiotic mixture. Sci. Rep. *9*, 4996.

Ridlon, J.M., Kang, D.J., and Hylemon, P.B. (2006). Bile salt biotransformations by human intestinal bacteria. J. Lipid Res. *47*, 241–259.

Sánchez-Villanueva, J.A., Rodríguez-Jorge, O., Ramírez-Pliego, O., Rosas Salgado, G., Abou-Jaoudé, W., Hernandez, C., Naldi, A., Thieffry, D., and Santana, M.A. (2019). Contribution of ROS and metabolic status to neonatal and adult CD8+ T cell activation. PLoS ONE *14*, e0226388.

Shi, Z., Li, G., Zhang, L., Ma, M., and Jia, W. (2020). *Klebsiella pneumoniae* infection inhibits autophagy by alveolar type II epithelial cells. Exp. Ther. Med. *20*, 3703–3708.

CellPress
OPEN ACCESS

Stein, A., and Bailey, S.M. (2013). Redox Biology of Hydrogen Sulfide: Implications for Physiology, Pathophysiology, and Pharmacology. Redox Biol. *1*, 32–39.

Turnbaugh, P.J., Ley, R.E., Mahowald, M.A., Magrini, V., Mardis, E.R., and Gordon, J.I. (2006). An obesity-associated gut microbiome with increased capacity for energy harvest. Nature *444*, 1027–1031.

Walker, A.W., Duncan, S.H., Louis, P., and Flint, H.J. (2014). Phylogeny, culturing, and metagenomics of the human gut microbiota. Trends Microbiol. *22*, 267–274.

Washio, J., Shimada, Y., Yamada, M., Sakamaki, R., and Takahashi, N. (2014). Effects of pH and lactate on hydrogen sulfide production by oral Veillonella spp. Appl. Environ. Microbiol. *80*, 4184–4188.

Wood, D.E., Lu, J., and Langmead, B. (2019). Improved metagenomic analysis with Kraken 2. Genome Biol. *20*, 257.

Yang, I., Corwin, E.J., Brennan, P.A., Jordan, S., Murphy, J.R., and Dunlop, A. (2016). The Infant Microbiome: Implications for Infant Health and Neurocognitive Development. Nurs. Res. *65*, 76–88.

Zhao, Y., Hu, Q., Cheng, F., Su, N., Wang, A., Zou, Y., Hu, H., Chen, X., Zhou, H.-M., Huang, X., et al. (2015). SoNar, a Highly Responsive NAD+/NADH Sensor, Allows High-Throughput Metabolic Screening of Anti-tumor Agents. Cell Metab. *21*, 777–789.

Zheng, Q., Li, Z., Zhou, S., Zhang, Q., Zhou, L., Fu, X., Yang, L., Ma, Y., and Hao, X. (2017). Heparin-binding Hemagglutinin of *Mycobacterium tuberculosis* Is an Inhibitor of Autophagy. Front. Cell. Infect. Microbiol. *7*, 33.

Zhirnov, O.P., and Klenk, H.D. (2013). Influenza A virus proteins NS1 and hemagglutinin along with M2 are involved in stimulation of autophagy in infected cells. J. Virol. *87*, 13107–13114.

Zhu, W., Lomsadze, A., and Borodovsky, M. (2010). Ab initio gene identification in metagenomic sequences. Nucleic Acids Res. *38*, e132.

Zimmermann, S., Pfannkuch, L., Al-Zeer, M.A., Bartfeld, S., Koch, M., Liu, J., Rechner, C., Soerensen, M., Sokolova, O., Zamyatina, A., et al. (2017). ALPK1- and TIFA-Dependent Innate Immune Response Triggered by the Helicobacter pylori Type IV Secretion System. Cell Rep. *20*, 2384–2395.

Zuo, H.-J., Xie, Z.-M., Zhang, W.-W., Li, Y.-R., Wang, W., Ding, X.-B., and Pei, X.-F. (2011). Gut bacteria alteration in obese people and its relationship with gene polymorphism. World J. Gastroenterol. *17*, 1076–1081.

STAR★METHODS

KEY RESOURCES TABLE

REAGENT or RESOURCE	SOURCE	IDENTIFIER
Bacterial and virus strains		
Escherichia coli EC100	Lucigen	Cat#EC10010
T7 Express *Escherichia coli*	New England Biolabs	Cat#C2566I
Chemicals, peptides, and recombinant proteins		
Pyruvate	Thermo Fisher Scientific	11360070
Hydrogen Sulfide	LabChem	LC154701
L-Cysteine	Sigma Aldrich	168149-500G
DL-Homocysteine	Sigma Aldrich	H4628-1G
Critical commercial assays		
End-it DNA End-Repair kit	Lucigen	Cat#ER0720
Fast-Link DNA Ligation kit	Lucigen	Cat#LK0750H
MaxPlax Lambda Packaging Extracts	Lucigen	Cat#MP5105
EZ-Tn5 insertion kit	Lucigen	Cat#EZI982K
Deposited data		
Genomic DNA insert sequences from cosmids giving hits in this study	This study	GenBank: MW341117-MW341204
Experimental models: Cell lines		
HEK293 NF-kB reporter	Cohen et al., 2015	N/A
HEK293 LC3 reporter	This study	N/A
NCI-H1299 SoNar reporter	This study	N/A
Oligonucleotides		
See Table S4 for oligonucleotide sequences		N/A
Recombinant DNA		
pJWC1	Craig et al., 2009	N/A
pCDNA3.1-SoNar	Yi Yang	N/A
pEGFP-LC3	Addgene	RRID:Addgene_24920
Software and algorithms		
Adobe Illustrator 2020	Adobe Inc.	N/A
GraphPad Prism 9	GraphPad Software	https://www.graphpad.com/scientific-software/prism/
Geneious Prime	Biomatters	https://www.geneious.com/prime/
seqtk		https://github.com/lh3/seqtk
Kraken2	Wood et al., 2019	https://ccb.jhu.edu/software/kraken2/
Bracken	Lu et al., 2017	https://ccb.jhu.edu/software/bracken/
MetaXpress	Molecular Devices	https://www.moleculardevices.com/products/cellular-imaging-systems/acquisition-and-analysis-software/metaxpress#gref
Other		
Synergy Neo plate reader	BioTek	N/A
ImageXpress XLS Widefield High Content microscope	Molecular Devices	N/A
TECAN Freedom EVO 150	Tecan	N/A
Illumina Miseq System	Illumina	RRID:SCR_016379
Thermo Multidrop Combi	Thermo Fisher	N/A
Waters Acquity HPLC	Waters	N/A

CellPress
OPEN ACCESS

RESOURCE AVAILABILITY

Lead contact

Further information and requests for resources and reagents should be directed to and will be fulfilled by the Lead Contact, Sean F. Brady (sbrady@rockefeller.edu).

Materials availability

The stably integrated GFP-LC3 and SoNar reporter cell lines described here are available upon request.

Data and code availability

The sequences of cosmids containing metagenomic DNA that produces effectors with activity against NF-kB, LC3 or SoNar reporter cell lines have been deposited in GenBank (Accession MW341117-MW341204). All other data needed to evaluate the conclusions in the paper are present in the paper or the Supplemental information.

This paper does not report original code.

Any additional information required to reanalyze the data reported in this paper is available from the lead contact upon request.

EXPERIMENTAL MODEL AND SUBJECT DETAILS

Cell lines and culture conditions

E. coli EC100 and T7 express (NEB) were grown in LB media supplemented with either tetracycline (15 μg/mL) or kanamycin (50 μg/mL) when appropriate. All cultures were grown at 37°C with shaking (200 rpm).

The NF-κB reporter cell line has been described previously (Cohen et al., 2015; Estrela et al., 2019) and consists of HEK293T cells stably transfected with a construct containing a minimal cytomegalovirus (CMV) promoter and four copies of the NF-κB transcriptional response element controlling expression of GFP. The LC3 reporter cell line consists of HEK293 cells stably transfected with pEGFP-LC3, which is under the control of the CMV promoter. GFP-LC3 is a standard marker for the autophagosome, the characteristic compartment of autophagy. HEK293 cells are originally derived from human embryonic kidney cells taken from an aborted female fetus. The SoNar reporter cell line consists of NCI-H1299 cells stably transfected with pCDNA3.1-SoNar (generously provided by Dr. Yi Yang) (Zhao et al., 2015) . NCI-H1299 cells are originally derived from human non-small cell lung cancer cells taken from a 43 years old adult Caucasian male. The SoNar construct is a fusion of cpYFP and the NADH-binding domain of T-Rex. Binding of NAD^+ or NADH induces unique conformational changes to T-Rex that alter the excitation spectrum of the T-Rex-cpYFP fusion. Upon NAD^+ binding, the excitation maximum is at 485 nm, while upon NADH binding it is at 420 nm (the emission maximum is unchanged at 520 nm). This makes the ratio of fluorescence at these wavelengths a measure of the intracellular ratio of NAD^+ to NADH. When the ratio of NADH to NAD^+ increases, so does the ratio of F_{420} to F_{485} and vice versa, providing a way of measuring intracellular redox potential.

NF-κB and LC3 cell lines were routinely cultured at 37°C with 5% CO_2 in Dulbecco's modified Eagle's medium (DMEM) supplemented with 10% fetal bovine serum (FBS), 200 mM L-glutamine, 100 U/mL penicillin, 100 U/mL streptomycin and phenol red (15.9 mg/L). SoNar cells were grown in RPMI-1640 medium (ATCC 30-2001) supplemented with 10% FBS. For assays, 1 mL of cells prepared from a single large culture and frozen in growth media with 10% DMSO were thawed, media replaced at 24 h, and grown to 85% confluence. Cells were trypsinized, counted (Countess; Invitrogen), and diluted in medium without phenol red, before seeding 25 μL into cell culture-grade clear-bottom, black-sided 384-well plates (Corning, New York, NY). NF-κB cells were seeded at 2500 cells/well, LC3 at 3500 cells/well, and SoNar at 1000 cells/well.

METHOD DETAILS

Metagenomic library construction

Stool samples were collected from volunteers with the assistance of the Rockefeller University Hospital Center for Clinical and Translational Science (Rockefeller IRB # SBR-0962). Infant stool samples were collected from the diapers of healthy infants belonging to two age groups, and metagenomic DNA from the stool was isolated using techniques adapted from soil environmental DNA extraction (Brady, 2007). Approximately 1-1.5 g of stool was suspended in 10 mL of phosphate-buffered saline (PBS), homogenized under vortex for 2 min, mixed with 20 mL of prewarmed (70°C) 1.5x lysis buffer (150 mM Tris-HCl, 150 mM Na-EDTA, 2.25 M NaCl, 1.5% cetrimonium bromide, 3% SDS, pH 8.0) and incubated at 70°C for 2 h with mixing by inversion every 30 min. Samples were centrifuged for 30 min (5000x g) at 4°C to pellet insoluble matter. The supernatant was centrifuged again, transferred to a new tube and crude DNA precipitated by addition of 0.7 vol isopropanol. DNA was washed with 70% EtOH and dried before resuspension in buffer TE (10 mM Tris-HCl, 1 mM Na-EDTA, pH 8.0). High-molecular-weight DNA was isolated by preparative (0.7% agarose) gel electrophoresis (3 h, 100 V/cm), excision of gel slice containing DNA of > 20 kbp, and electroelution (100 V/cm, 2h) of DNA from this slice. Isolated metagenomic DNA from each infant was sequenced using an Illumina MiSeq next-gen sequencer and samples were then pooled for library construction. Purified high-molecular-weight DNA was blunt-ended (End-It DNA End-Repair kit; Lucigen, Middleton, WI), ligated (Fast-Link DNA Ligation kit; Lucigen) into ScaI-digested

pJWC1 cosmid vector (Craig et al., 2009), packaged into lambda phage (MaxPlax Lambda Packaging Extracts; Lucigen) and transformed into *E. coli* EC100 (Lucigen). Clones were selected by plating transformed *E. coli* on LB agar plates containing 15 μg/mL tetracycline and 10% sucrose. A total of 37,772 individual colonies were robotically arrayed into 142 384-well plates and stored at −80°C as glycerol stocks, with clones transformed with empty pJWC1 vector included in three separate columns of each plate as a negative control.

16S sequencing and analysis

16S rDNA was PCR-amplified from purified infant microbiome DNA with barcoded forward and reverse primers (16S annealing regions: forward 5′-GTGYCAGCMGCCGCGGTAA, reverse 5′-GGACTACNVGGGTWTCTAAT) using the following PCR protocol: 95°C for 5 min, 35 cycles of [95°C for 30 s, 55°C for 30 s, 72°C for 45 s], 72°C for 8 min. PCR samples were run on a 2% agarose gel and the appropriately-sized bands excised and gel-purified before being subjected to a second round of PCR (95°C for 5 min, 9 cycles of [95°C for 30 s, 70°C for 30 s, 72°C for 1 min], 72°C for 8 min) to add Illumina adaptors, followed by MiSeq sequencing. Raw Illumina reads were subjected to quality control using seqtk with the trimfq option. Trimmed data were analyzed using Kraken2 (Wood et al., 2019) to assign taxonomy followed by Bracken (Lu et al., 2017) to compute relative abundances of taxa based on number of reads (Bracken abundance analysis given in Table S1).

Generation and high-throughput screening of sterile spent culture broth filtrates

Arrayed metagenomic clones were pin transferred into sterile deep-well 384-well microplates containing 150 μL of LB + 15 μg/mL tetracycline per well. Arrayed clones were grown for 18 h at 37°C (shaking at 200 rpm), and then subcultured (40 nL) into microplates containing LB + 15 μg/mL tetracycline. These plates were incubated at 30°C without shaking for 4 d. Mature culture plates were centrifuged at 1,600x *g* for 15 min to pellet the *E. coli*. Using an automated liquid-handling system (Tecan EVO, Tecan, Männedorf, Switzerland), 80 μL of supernatant was transferred to a 0.2-μm membrane AcroPrep 384-well filter plate (Pall, Port Washington, NY) and sterile spent culture broth filtrate collected by centrifugation (800x *g*, 2 min). Where applicable, sterile filtrate was further passed through a 3-kDa Amicon centrifugal filter unit (Millipore Sigma) according to the manufacturer's instructions.

Automated liquid-handling (Tecan EVO) was then used to transfer 25 μL of sterile spent culture broth filtrate onto mammalian cells seeded as described above. After 24 h of incubation for SoNar cells, fluorescence was measured on a plate reader (Synergy Neo plate reader, BioTek, Winooski, VT) and the ratio at 420 nm to 485 nm (F_{420}/F_{485}) used as a measure of cellular redox state. After 24 h of incubation for NF-κB or 2 h of incubation for LC3, 10 μL of a phosphate-buffered saline (PBS) solution containing nuclear staining (2 μg/mL Hoechst 33342) and dead cell marker (6 μg/mL propidium iodide) was added to each well using a Multidrop instrument (Thermo Fisher Scientific, Waltham, MA). Images were taken with a 10x objective using an ImageXpress XLS Widefield High Content Microscope (Molecular Devices, San Jose, CA) with the fluorescent filters Texas red (excitation, 562 nm [562_{ex}]; emission, 624 nm [624_{em}]) for propidium iodide (PI), fluorescein isothiocyanate (FITC; 482_{ex} and 536_{em}) for GFP, and 4′,6′-diamidino-2-phenylindole (DAPI; 377_{ex} and 447_{em}) for Hoechst 33342 and analyzed using an automated custom module in MetaXpress software (Molecular Devices). MetaXpress automated microscopy analysis scripts were written in-house. Cell death was assessed as the ratio of PI-stained cells to total Hoechst-stained nuclei in each well. NF-κB activation was measured as the ratio of live GFP-expressing cells to total live cells in each well. LC3 activation was measured as the number of GFP-LC3 granules per live cell in each well. Results were normalized to a set of negative-control wells in each plate (*E. coli* host transformed with empty pJWC1 vector) and expressed as *Z*-scores. Z score = $(MEAN_{Test} - MEAN_{control})/(SD_{control})$. Clones with a Z-score greater than 3 were re-assayed in quadruplicate using the same protocol and determined to be validated if 3 out of 4 wells consistently showed a Z-score > 3.

Sequence annotation of inserts from bioactive clones and identification of effector genes by transposon mutagenesis

Cosmid DNA was obtained from each bioactive clone using a Monarch Plasmid Miniprep kit (NEB, Ipswich, MA), and sequenced on an Illumina MiSeq next-generation sequencer. *E. coli* genomic DNA and pJWC1 were computationally filtered out and remaining reads were assembled using Geneious (Biomatters, San Diego, CA, USA). Cosmids were mapped to each other using Geneious "Map to Reference" function using default settings and assigned to the same candidate gene family of clones if > 5 kbp of DNA overlapped. The longest contig in each cosmid was submitted to a blastn search to determine the insert origin. ORFs were predicted using Metagenemark (Zhu et al., 2010) and searched against the NCBI nonredundant (nr) database using blastx. Gene tables are given in Table S4. Cosmid DNA from selected bioactive clones was mutagenized using the EZ-Tn*5* < KAN-2 > insertion kit (Lucigen) according to the manufacturer's instructions, desalted using agarose gel tubes, and transformed into electrocompetent *E. coli* EC100 cells. Mutants were selected on LB agar using 50 μg/mL kanamycin and 15 μg/mL tetracycline, and single colonies were inoculated and assayed in quadruplicate in the different reporter systems as described above, with the non-mutagenized clone as a positive control. Inactive mutants were identified, and the transposon insertion locations were determined by Sanger sequencing using transposon-specific sequencing primers KAN-2 FP-1 or KAN-2 RP-1 (Lucigen).

Identification of effector genes in patient metagenomes

Using a BLASTx search with default parameters, a representative effector gene from each gene family identified by transposon mutagenesis was aligned against a non-redundant catalog of bacterial proteins from the HMP (HMGC, https://www.hmpdacc.

org/hmp/hmgc2/) derived from patient metagenomes. Protein alignments with > 90% sequence identity and 70% coverage were considered "matches" to the queried sequence.

Broad proteobacterial expression of MATE-family transporters

Electrocompetent *Caulobacter vibroides* C15, *Agrobacterium tumefaciens* LBA4404, and *Burkholderia graminis* C4D1M were prepared and transformed (as described previously (Craig et al., 2010)) with the cosmid N044. Single colonies of each strain were grown overnight (30°C, 200 rpm) in their appropriate medium (*C. vibroides*: ATCC36, 4 μg/mL tetracycline; *A. tumefaciens*: yeast-mannitol, 5 μg/mL tetracycline; *B. graminis*: LB, 20 μg/mL tetracycline) prior to inoculating 50 mL of fresh medium with the appropriate tetracycline concentration. These cultures were grown for 24 h (30°C, 200 rpm) and culture supernatant was collected by centrifugation (4,000 x g, 20 min, 4°C).

NF-kB reporter cells were seeded in black 96 well, clear bottom plates (Corning) at a density of 2,000 cells/well and allowed to attach overnight. Culture medium was aspirated and replaced with fresh culture medium (90 μL) and proteobacterial supernatant (10 μL). Following 24 h incubation, NF-kB activation was measured as described above. Samples were tested in triplicate.

LC-MS analysis of N-acetyltransferase-containing clones

E. coli containing the Cbeg12 cosmid (positive control)(Cohen et al., 2015), pJWC1 cosmid vector (negative control) or *N*-acetyltransferase-containing cosmids from this study were grown in LB medium supplemented with 15 μg/ml tetracycline for 4 days at 30°C and 250 rpm. Cultures were extracted 1:1 with ethyl acetate and dried *in vacuo*. Extracts were run on a Waters Acquity HPLC with a 10 × 250 mm Waters XBridge C18 column using a gradient of solvent A (water + 0.1% formic acid) and solvent B (acetonitrile + 0.1% formic acid), starting at 90% A: 10% B (0 - 0.9 min) ramping to 100% B over 3.6 min, holding at 100% B for 1.8 min and returning to 90% A: 10% B by 6.30 min. This LC system was coupled to a Waters Acquity QDa Mass Detector run in negative ion mode with a mass range of 50 to 1000 Da.

NF-kB activation by pure commendamide

NF-kB reporter cells were seeded in black 96 well, clear bottom plates (Corning) at a density of 2,000 cells/well and allowed to attach overnight. Culture medium was aspirated and replaced with fresh culture medium containing commendamide (Cayman Chemical) at the desired concentration. Following 24 h incubation, NF-kB activation was measured as described above. All concentrations were tested in triplicate.

Subcloning and inducible expression of effector genes

Primers with selected restriction sites (Table S5) were designed to PCR-amplify the nucleotide sequence of effector genes of interest from the pJWC1 insert template cosmid with Q5 High-Fidelity DNA polymerase (NEB) according to the manufacturer's protocol. PCR products were gel purified and digested with indicated restriction enzymes (NEB), ligated using T4 DNA ligase (NEB) into pET28c vector digested with appropriate restriction enzymes and dephosphorylated. The ligation reaction was transformed into electrocompetent T7 express *E. coli* (NEB). Clones containing the desired insert were confirmed by Sanger sequencing using T7 and T7-term primers. Clones were cultured in LB medium in the presence of 50 μg/mL kanamycin and, upon reaching an optical density at 580 nm (OD_{580}) of 0.6, induced with 500 μM isopropyl-β-D-thiogalactopyranoside (IPTG) for 20 h at 18°C. Sterile spent broth collected after this period was tested for activity using the assays as described above, or mixed with 2x SDS-PAGE loading buffer (Biorad), heated for 10 min and run on a 12% SDS-PAGE gel to investigate of the presence of secreted proteins.

LC3 activation by L001-A

The putative secretion sequence of the L001-A gene product was predicted using SignalP 5.0 (Almagro Armenteros et al., 2019). *L001-A* encoding the cleaved product was restriction cloned into pET28c using NheI and XhoI (NEB) to contain an N-terminal His tag for purification (Table S5). This vector was transformed into electrocompetent *E. coli* BL21(DE3), and a single colony was grown overnight in LB medium (50 μg/mL kanamycin) at 37°C with shaking (200 rpm). This overnight culture was used to inoculate 1 L of fresh LB medium (50 μg/mL kanamycin). Upon reaching an OD_{600} of 0.6, protein expression was induced by the addition of IPTG (500 μM). Expression continued for 3 h at 37°C with shaking, at which point cells were harvested by centrifugation (4,000 x g, 20 min) and the cell pellets were stored at −20°C. Cells were resuspended in lysis buffer (50 mM Na_2HPO_4, 300 mM NaCl, 10 mM imidazole, pH 8.0), lysed by sonication (10 cycles of 30 s on and off, 4°C) and centrifuged to pellet cellular debris (4000 x g, 30 min). Supernatant was collected, mixed with Ni-NTA resin (QIAGEN) and incubated for 2 h at 4°C. This slurry was decanted into a gravity column, washed twice with 8 column volumes of wash buffer (50 mM Na_2HPO_4, 300 mM NaCl, 50 mM imidazole, pH 8.0), and eluted 4 times with 1 column volume of elution buffer (50 mM Na_2HPO_4, 300 mM NaCl, 500 mM imidazole, pH 8.0). Protein was quantified using a BCA Protein Assay kit (Pierce). Purified protein was dialyzed overnight against PBS (15 mM, Na_2HPO_4, 137 mM NaCl, 2.7 mM KCl, pH 7.4) at 4°C.

LC3 reporter cells were seeded in black 96 well, clear bottom plates (Corning) at a density of 2,000 cells/well and allowed to attach overnight. Culture medium was aspirated and replaced with fresh culture medium containing purified L001-A at the desired concentration. Following 2 h incubation, LC3 activity was measured as described above. All concentrations were tested in triplicate.

QUANTIFICATION AND STATISTICAL ANALYSIS

Graphs were plotted and statistics calculated using GraphPad Prism 9 (San Diego, CA, USA). Statistical analyses are described in the figure legends and Results section. Statistical analysis was performed using a one-way ANOVA with a Dunnett's post hoc test comparing each group to the empty vector control (** $p < 0.01$, ****$p < 0.0001$).

Cell Reports, Volume *36*

Supplemental information

Multiplexed functional metagenomic analysis of the infant microbiome identifies effectors of NF-κB, autophagy, and cellular redox state

Frank J. Piscotta, Shawn T. Whitfield, Toshiki G. Nakashige, Andreia B. Estrela, Thahmina Ali, and Sean F. Brady

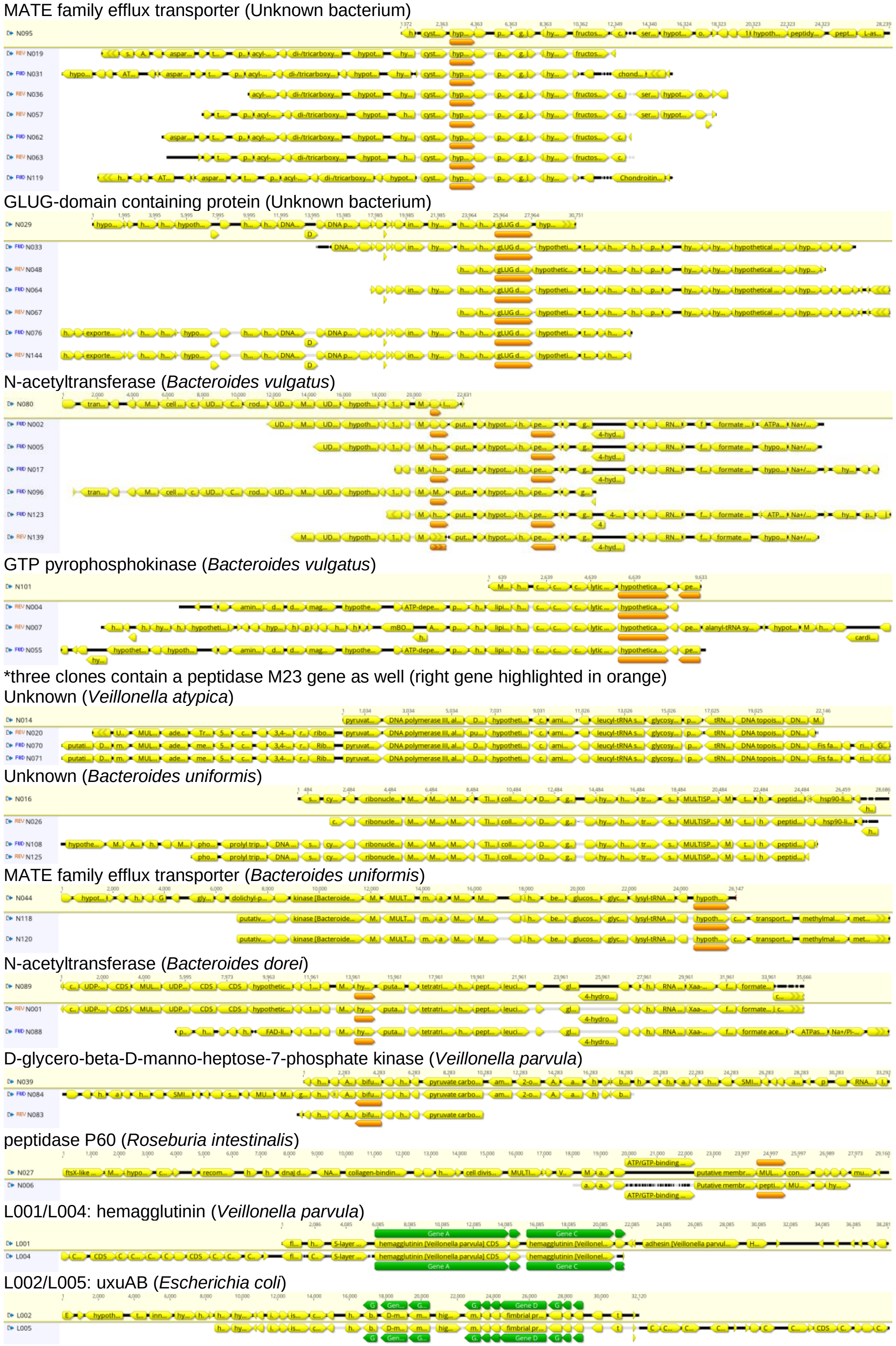

Figure S1 Overlapping genomic regions obtained by alignment of sequenced bioactive cosmids. Orange highlighting indicates genes with known NF-κB-inducing activity or genes suggested to be bioactive by transposon mutagenesis. Green highlighting indicates prospective autophagy-inducing genes selected for subcloning in Figure 4. Related to Figure 3C-D, Figure 4A.

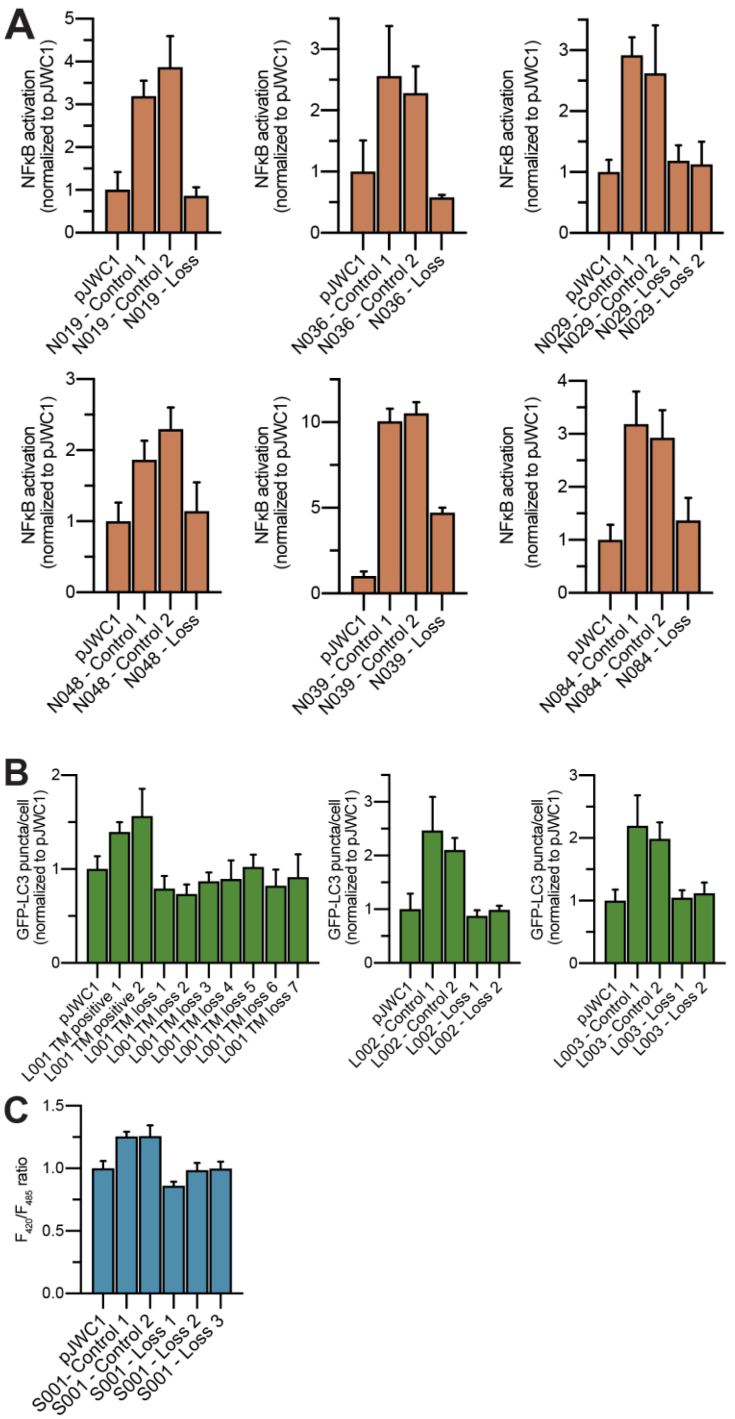

Figure S2 Transposon mutagenesis of selected cosmid clones for identification of genes causing activation of NF-κB, LC3 or SoNar reporter lines. All data are represented as mean ± SD, n=4. (A) Transposon mutagenesis of NF-κB active clones. “Loss” samples indicate where transposon insertions resulted in loss of activity, as confirmed by Sanger sequencing. Related to Figure 3D. (B) Transposon mutagenesis of autophagy active clones. “Loss” samples indicate where transposon insertions resulted in loss of activity, as confirmed by Sanger sequencing. Related to Figure 4A. (C) Transposon mutagenesis of SoNar active clones. “Loss” samples indicate where transposon insertions resulted in loss of activity, as confirmed by Sanger sequencing. Related to Figure 5A.

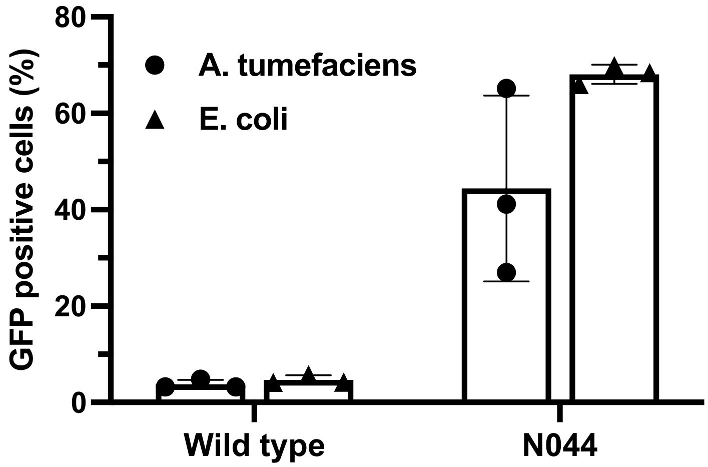

Figure S3. ***A. tumefaciens*** **(circle) and** ***E. coli*** **(triangle) transformed with the MATE family exporter-carrying cosmid N044 activate NF-kB reporter cells.** Both wild-type strains and strains transformed with N044 were grown at 30 ° C for 2 d and supernatants collected by centrifugation were applied to NF-kB reporter cells at a 1:5 dilution. All data are represented as mean ± SD, n=3. Related to Figure 3.

N-acetyltransferase (*Bacteroides vulgatus*)

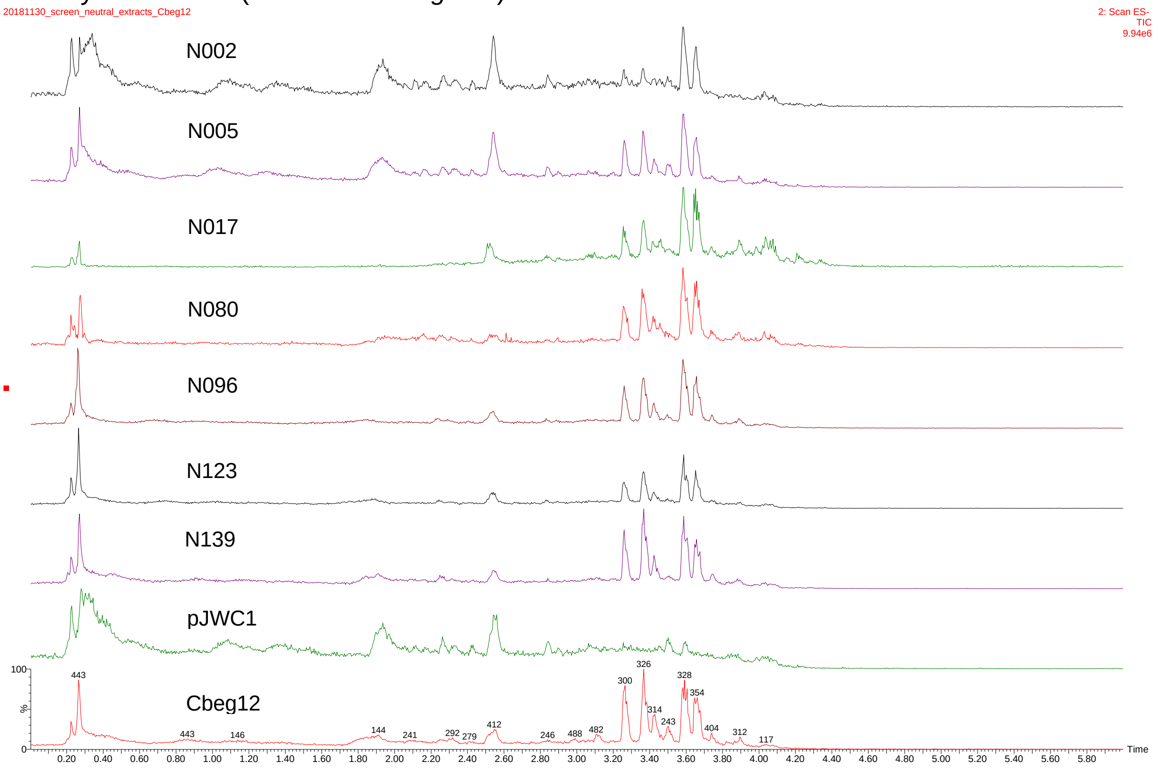

N-acetyltransferase (*Bacteroides dorei*)

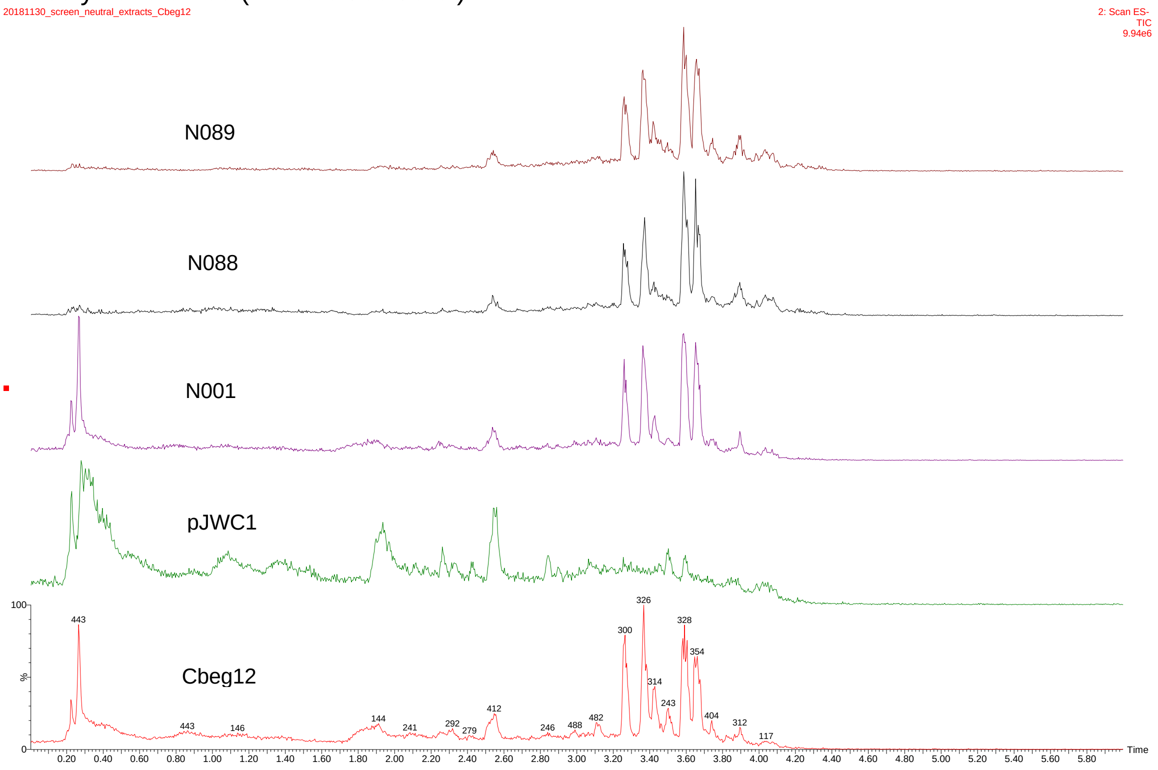

Figure S4 LCMS traces of extracts from commendamide-producing N-acetyltransferase clones compared to pJWC1 empty control and Cbeg12 positive (commendamide-producing) control. Commendamide-related products of masses of 300, 325, 328 and 354 are found in all positive samples and Cbeg12 but not pJWC1. Related to Figure 3E.
